# Comprehensive benchmarking and explainable machine learning analysis of EEG imagery activity recognition

**DOI:** 10.1038/s41598-026-50997-y

**Published:** 2026-05-16

**Authors:** Md. Julkar Nain Siam, Tanvir Ahsan Showrov, Md. Sakir Hossain, Najmus Shakif Ayaan, S. M. Sadakatul Bari, Faisal Tariq, ASM Ashraf Mahmud

**Affiliations:** 1 Department of Avionics Engineering, Aviation and Aerospace University, Bangladesh, Dhaka, 1215 Bangladesh; 2 Department of Computer Science Engineering, BRAC University, Dhaka, 1212 Bangladesh; 3 James Watt School of Engineering, University of Glasgow, Glasgow, G12 8QQ UK; 4 School of Computing, Engineering and Physical Sciences, University of the West of Scotland, High St, Paisley, PA1 2BE UK

**Keywords:** Brain–computer interface, Electroencephalogram, Extra tree, Bayesian hyperparameter tuning, Computational biology and bioinformatics, Engineering, Mathematics and computing, Neuroscience

## Abstract

Motor imagery (MI)-based brain–computer interfaces (BCIs) enable users to control external devices using EEG signals, offering great potential in assistive and rehabilitation technologies. However, MI recognition remains challenging due to EEG’s low signal-to-noise ratio (SNR), inter-subject variability, and complex spatiotemporal patterns. Existing approaches often suffer from limited accuracy, high computational cost, and poor interpretability. In response to these challenges, we present the first comprehensive benchmarking of the publicly available EEG-hand movement (EEG-HM) dataset. Our study aims to establish a standardized performance baseline, guide the selection of optimal models by jointly considering accuracy, prediction time, and explainability, and ultimately accelerate progress in MI-BCI development. We have proposed a two-stage optimization of machine learning models that employs both feature selection and hyperparameter tuning. We exploit five feature selection algorithms for selecting the best set of EEG electrodes and frequency bands, while Bayesian optimization is exploited for machine learning model optimization through hyperparameter tuning. Furthermore, to validate the neurophysiological basis of our model’s decisions, we leverage explainable AI (XAI) algorithms—LIME and SHAP—quantifying the contributions of specific EEG electrodes and frequency bands to interpret its decision-making process. Through extensive simulations, the proposed two-stage optimization of the machine learning model demonstrates a superior performance in terms of accuracy, precision, and recall. This method outperforms the existing methods by 21.47% in accuracy with competitive prediction time. Its performance is further evaluated on the PhysioNet MI dataset, achieving a 4.67% accuracy improvement over state-of-the-art methods. Through LIME and SHAP, we provide the local and global explanations for no activity, left-hand, and right-hand imagery movements. Additionally, we analyze how various EEG frequency bands and electrode locations interact during the performance of different motor imagery hand movements.

## Introduction

The brain–computer interface (BCI) system has become a revolutionary technology, enabling direct communication between the human brain and external electronic devices by interpreting brain waves into commands, bypassing traditional motor pathways. For severe motor-impaired people caused by stroke, amyotrophic lateral sclerosis, or spinal injuries, BCI plays an important role to improve the quality of their lives. It can be used for controlling wheelchairs, prosthetic arms/limbs, and other assistive devices. BCI is implemented based on the brain waves generated due to various activities of the subjects. However, heavily motor-impaired person cannot move their certain body parts. In this case, the brain signal due to the motor imagery (MI) is collected, where the motor imagery is the mental process of imagining a movement of a body part, such as left or right hand, fingers, tongue, and so on, without physically moving it. Among the BCI paradigms, MI-based BCI is the most prominent because it depends on endogenous brain activity and does not require an additional stimuli^1^. Brain waves are captured using both invasive and non-invasive methods. Of them, the invasive methods, such as intra-cortical electrodes and electrocorticography (ECoG), need implantable devices directly inside the brain or inside the scalp to capture signals. Although the resulting signal has a high signal-to-noise ratio (SNR), it has significant risks and ethical concerns. On the other hand, various devices are attached on top of the scalp to record neuronal signals in non-invasive methods. Various technologies, such as functional magnetic resonance imaging (fMRI), electroencephalography (EEG), and magnetoencephalography (MEG), use this approach of signal collection. The non-invasive methods are easy to apply and have no potential risk^2^. However, the resulting signals suffer from low SNR. One of the most popular, easiest, and low-cost technologies among this group is EEG. In capturing EEG signals, various number of electrodes are placed on the scalp in a fixed spatial pattern depending on the EEG headset. Due to the high temporal resolution and non-invasive nature, EEG-based BCIs are employed in a wide-variety of applications such as emotion recognition^3^, person identification^4^, driver fatigue detection^5^, stroke rehabilitation^6^, activity recognition^7^, and exoskeleton control^8^. However, EEG signals are naturally weak, easily disrupted by interference, and vulnerable to noise, leading to a low SNR. Furthermore, EEG signals vary significantly for the same subject for the same activity due to the non-stationary nature of this signal. The signal analysis becomes more complicated due to the complex correlation among various channels of EEG^9^. Since a BCI translates a subject’s intention decoded from the EEG signal to a specific action through an external device, the performance of the BCI largely depends on the detection accuracy of the subject’s intention from the EEG signals. For this reason, motor imagery activity recognition is the central challenge in developing an effective MI-BCI.

Among the applications of EEG, activity recognition is considered as a vital method for helping disabled people lead a better life. By moving various parts of their body, such as hands, legs, tongue, fingers, and so on, they can control assistive devices for household work. The effectiveness of the assistive devices largely depends on the detection of the user’s activity. The movement of the body parts can be motor executed or motor imagery. A considerable effort is devoted to date in detecting EEG signal excited due to the motor executed activity^10–14^. For example, EEG is employed for hand movement^11^, fist and feet movement^12^, hand and wrist movement^14^, and level ground walking, going down stairs, up stairs, up ramps, and down ramps^13^. However, motor disabled people cannot use the assistive devices that are controlled by motor execution activity. To enable them to control assistive devices, the devices must be controlled from the EEG signal of motor imagery activity. However, a considerable effort is also devoted to developing motor imagery EEG-based assistive devices. Researchers develop detection methods for various motor imagery activities, such as lower limb movement^15^, finger movement^16^, right-hand and right-foot movement^17^, left-hand and right-hand movement^18,19^, left-hand, right-hand, feet, and tongue movement^20^. However, most of the works suffer from two limitations: limited accuracy and impractical experimental settings. For instance, the detection method for right-hand and left-hand motor imagery movement^18,19^ fails to achieve a good detection accuracy. On the other hand, most of them do not consider real-world artifacts, such as eye blinking, head movement, muscle activity, and environmental electrical noise, which can significantly affect the quality and reliability of EEG signals. Due to this reason, the performance of these methods obtained in laboratory settings cannot be obtained in real-world applications. Moreover, while the performance of machine learning models for motor imagery recognition heavily depends on dataset quality, the EEG-hand movement (EEG-HM) dataset introduced in^19,21^ remains largely underexplored. To date, only a single study^19^ has utilized this dataset for motor imagery activity recognition, and the proposed model achieves limited accuracy. Consequently, the full potential of EEG-HM has yet to be realized through systematic and comprehensive analysis.

In the MI-BCI community, benchmark MI-EEG datasets such as the PhysioNet EEG Motor Movement/Imagery Dataset (derived from the BCI2000 platform) and the BCI Competition III – Dataset IVa have been widely adopted. Prior studies conducted on these datasets primarily focused on spectral feature extraction followed by spatial filtering (e.g., Common Spatial Patterns) and classical classifiers such as linear discriminant analysis (LDA) or support vector machine (SVM)^22,23^. However, they did not integrate systematic feature selection, Bayesian hyperparameter optimization, and explainable AI into a unified benchmarking framework. On the other hand, our study provides the first comprehensive benchmarking of the EEG-HM dataset by applying these elements together to provide a more exhaustive evaluation of machine learning approaches for motor imagery classification.

In this paper, we propose a motor imagery activity recognition method for detecting right-hand and left-hand movement. We utilize a dataset of extracted EEG features in which artifact-related conditions are inherently present as part of the original data acquisition protocol, reflecting realistic recording scenarios.

- Conducted the first comprehensive benchmarking of the publicly available EEG motor imagery EEG-HM^19^ dataset, systematically evaluating 11 machine learning classifiers combined with 5 feature selection methods under a subject-dependent validation framework, all optimized via Bayesian hyperparameter tuning.
- Proposed a high-performing pipeline combining recursive feature elimination (RFE) feature selection method and extra tree classifier that significantly outperforms the existing work, achieving 21.47% higher accuracy, setting a new performance benchmark.
- Validated the performance of the proposed optimized extra tree with RFE pipeline on the independent PhysioNet EEG motor imagery dataset, achieving 4.67% higher accuracy than state-of-the-art methods, demonstrating consistent cross-dataset performance.
- Provided an in-depth explainable AI (XAI) analysis using model-agnostic and model-specific interpretation techniques to uncover decision patterns, feature importance, and neural correlates in EEG motor imagery activity recognition.

The rest of this paper is organized as follows. “Literature review” reviews related work on EEG-based motor imagery activity recognition. “Dataset” describes the EEG-HM dataset in detail. “Proposed methods” presents the proposed machine learning pipeline, feature selection strategies, and explainability methods. “Experimental results and analysis” defines evaluation metrics, reports comprehensive experimental results, and provides in-depth analysis. Finally, “Conclusion” concludes the paper and discusses future directions.

## Literature review

In^24^, the authors propose an auto-encoder (AE)-based method for person identification and activity recognition. The correlation coefficient matrix and auto-encoder are exploited for feature extraction, which is followed by the classification using extreme gradient boosting (XGBoost) classifier. Five activities are considered: eyes closed, focusing on left-hand, right-hand, both hands, and both feet. Both the private dataset and publicly available PhysioNet EEGMMIDB^23^ are used to train and test the proposed method. The AE and XGBoost combination provides better results, a 10% improvement in accuracy, compared to principal component analysis (PCA)+XGBoost, and PCA+AE+XGBoost. The authors in^25^ propose an explainable ML model for EEG-based activity recognition using a combination of three ML algorithms, random forest, gradient boosting, and XGBoost, and LIME framework to interpret the influence of EEG features. 20 features are selected using the SelectKBest. The dataset considers three activities such as resting state, motor tasks, and cognitive tasks. The random forest model provides the best performance with an accuracy of 80.33%.

A method for facial activities (e.g., jaw-clenching and eye movements) recognition is proposed in^10^ with three artifacts: reading, speaking loudly, and watching TV. The framework uses a combination of convolutional neural networks (CNN) and long short-term memory (LSTM) to classify EEG signals collected from a consumer-grade EEG wearable device. This model achieves an accuracy of 94.66% using two EEG channels (AF7 and AF8) and 84.53% with a single EEG channel (AF7). Another activity recognition method using a consumer-grade EEG device is proposed in^12^. The authors used an Ultracortex Mark IV EEG headset with 8 channels to record brain activity during three motor tasks: movements of foot, fists, and relaxation. A 15-layer CNN is used for classification. The highest accuracy of 97.4% is found for recognizing fist movements, while foot movement is recognized with 89.6% of accuracy. Relaxation is classified with 94.6% accuracy.

The upper-limb movement recognition using EEG signals is investigated in^11^. The authors propose a deep learning (DL) network that classifies rest and hand movements. The DL model is trained separately for each subject, and a majority voting method is applied during post-processing to enhance prediction accuracy. The DL network achieves over 80% accuracy with a 600ms window size, which further improves to over 90% accuracy using a majority voting scheme. In^13^, the author investigates the identification of locomotion activities using EEG signals to control prosthetic devices. The framework uses independent component analysis (ICA) for feature extraction and artifact removal. Five locomotion tasks are considered: walking on level ground, ascending stairs, descending stairs, ascending ramps, and descending ramps. EEG signals are filtered using a Butterworth band-pass filter. The random forest outperforms all other methods, achieving an overall accuracy of 93%. To enable control of bio-robotic applications like prosthetic limbs, various DL approaches are exploited in^14^ for classifying hand and wrist movements using EEG and EMG signals. The DL approaches include CNN, LSTM, and a CNN-LSTM hybrid model. EEG is recorded from above-elbow amputees performing five different wrist and hand movements (wrist supination, wrist pronation, hand close, hand open, and no movement). The LSTM model achieved the best performance with 95.2% accuracy. In^16^, the authors focus on recognizing finger movements using EEG signals to control upper limb prosthetics. Power spectral density (PSD) and logistic regression are used as the primary feature and classifier, respectively. The EEG signals are recorded from a right-handed, neurologically healthy participant performing specific finger movements. Logistic regression achieves the best accuracy, with a mean classification accuracy of 65% for finger movements.

In^15^, the authors propose an improved CNN model for motor imagery EEG signal recognition to improve human-computer interaction for patients with limb disabilities. The approach involves preprocessing EEG signals with short-time Fourier transform (STFT) and continuous Morlet wavelet transform (CMWT) to create a time-frequency matrix. The method achieves 93.24% accuracy. Two more motor imagery-based activity recognition methods are proposed in^26,27^ for BCI. In^26^, the movements of the left or right hand are recognized. Features are extracted by combining PCA and Fisher’s Linear Discriminant (FLD). The authors propose a hybrid Kernel extreme learning machine (H-KELM) for classification. The proposed classifier achieves a classification accuracy of 96.54%. In^27^, the authors apply EEG source imaging to map EEG onto cortical regions, enabling more precise spatial localization of motor imagery tasks compared to traditional sensor-based approaches. EEG is recorded from five subjects using a 64-channel SynAmpsRT system while subjects imagine complex right-hand movements. The overall classification accuracy reaches 82.2%, 12.7% higher than sensor-based methods.

A BCI-based intelligent wheelchair is developed by exploiting EEG for four motor imagery tasks^28^. EEG is preprocessed with a band-pass filter, and discrete Fourier transform (DFT) is applied for feature extraction. A multilayer perceptron (MLP) classifier is trained to recognize motor imagery tasks. Another EEG-based device controlling is proposed in^29^ where motor-imagery EEG is used for controlling home appliances, such as the speed control of blenders. EEG data is collected for real and imagined ball-grasping movements. This method achieves an average accuracy of 74.96%.

Yu et al. ^30^ propose a computer-aided broad learning EEG system (CABLES)—a fully automated, unified sequential framework with three novel modules to address the problem of domain-specific EEG classification methods lacking cross-discipline adaptability. The method is tested on seven public datasets. Feed-forward neural network (F-FNN) achieves the best accuracy of 99.1% for the motor imagery dataset. In^31^, the authors address the problem of lack of versatility and poor generalizability in existing MI-based BCI systems. They introduced a fully automated framework, tested on BCI Competition III IVa, IVb, and GigaDB datasets for both subject-specific (SS) and subject-independent (SI) settings. The combination of variational mode decomposition (VMD), logistic regression (LR), and cascade feed forward neural network (CFNN) reaches the best classification accuracy of 99.5% on SS on the BCI Competition III IVa dataset.

In real-world EEG MI-BCI systems where electrode placement varies, the authors in^32^ propose a novel electrode domain adaptation network (EDAN) architecture to minimize the domain gap across electrodes and achieve a reliable cross-electrode performance. The method significantly improves classification accuracy compared to baseline methods. Chen et al.^33^ introduce EEGProgress—a progressive convolution CNN framework to extract the topological spatial information of EEG signals from multi-scale levels, designed for both cross-subject and within-subject MI classification. The model provides 4.02% higher average accuracy over existing CNN-based models.

In^17,18,20^, the authors address the problem of classifying motor imagery tasks from EEG signals for BCI applications. A method is proposed that uses the common spatial pattern (CSP) for feature extraction and Gaussian Naive Bayes (GNB) for classification. Two feature selection techniques, minimum redundancy maximum relevance (mRMR) are employed for selecting features that are highly relevant to the target class, and Lasso regularization for refining the selection by enforcing sparsity. The study uses the BCI Competition IVa dataset. It achieves an accuracy of 95.47%.

The authors propose a neighborhood component analysis for feature selection combined with an SVM classifier^20^ for MI-based BCIs. The study uses publicly available datasets from BCI Competitions III and IV. The proposed method achieves an average classification accuracy of 92.2%. In^18^, the authors propose a deep learning framework combining a CNN and a variational autoencoder (VAE). The study uses the BCI Competition IV dataset 2b and a custom dataset with MI tasks involving left and right hand imagery. The CNN-VAE framework achieves a kappa value of 0.564 on the BCI Competition IV dataset 2b. In^34^, the authors address the classification of motor imagery EEG signals to improve the accuracy of BCI for applications such as assisting individuals with disabilities to control external devices, such as prosthetics and wheelchairs. A multilayer CNN feature fusion method is proposed. The study uses the BCI Competition IV-2a and High Gamma datasets. The proposed MCNN method achieves 75.7% accuracy on the BCI Competition IV-2a dataset and 95.4% accuracy on the High Gamma dataset.

## Dataset

In this section, we will describe the EEG dataset employed for EEG-based hand movement prediction^19^. In the data collection phase, the brain impulses were collected by an EMOTIVE EPOC+ 14 Channel EEG device consisting of 14 electrode channels: AF3, AF4, F7, F8, F3, F4, FC5, FC6, T7, T8, P7, P8, O1, and O2. Data were collected for three motor imagery tasks: neutral or no activity, left-hand, and right-hand motor imagery movement. For the collection of activity recognition data, the EEG is recorded in 6 acquisition cycles. During data collection of voluntary motor control (mental commands), a paradigm was set for each acquisition cycle in which certain additional activities, such as sitting with eyes open or eyes closed and squeezing a sensory stimulating ball with the dominant hand, were also performed. At the same time, moving the palm of the other hand up and down, snapping the fingers, opening and closing that hand was also done. These activities, in addition to the three motor activities desired, are done to make the activity recognition system more realistic so that it can detect motor activities in various scenarios. The dataset therefore includes multiple artifact-related conditions as part of the original EEG-HM acquisition protocol; however, these were not explicitly quantified or controlled in the present study. In each acquisition cycle, the participants were shown a picture of a right arrow, a left arrow, and a circle to make voluntary motor activities such as right-hand movements, left-hand movements, and no motor activity, respectively. In each motor activity, EEG signals were collected.

EEG data is extracted from 14 channels at a sampling frequency of 128 Hz, in the form of a $128\times 14$ matrix, using a 16-bit analog-to-digital converter. Then, the Fourier transform of the EEG data is computed over a frequency range of 0-30 Hz. These Fourier-transform-based band features were already provided in the public EEG-HM CSV files. In the frequency domain, the value *w*(*i*, *j*) represents the value of the *j*th channel for *i*th frequency. Based on these values, the presence of four EEG frequency bands, such as delta (0–4Hz), theta (4–8Hz), alpha (8–12Hz), and beta (12–30Hz), is identified, with each band representing some momentary status of the brain. Then, the standard deviation and arithmetic average of each wave are computed, which gives a matrix of $14\times 4\times 2$. Each element of the matrix represents one of the 14 channels’ one of the four bands’ standard deviation or mean. The final dataset for each participant contains $14\times 4\times 2=112$ features (e.g., AF3 alpha std, F7 delta m, FC5 Beta m, etc.) with the ‘Class’ representing the motor activities performed during each cycle. The present study uses these precomputed 112 features as the input feature space for benchmarking, feature selection, hyperparameter tuning, and explainability analysis. The no-activity, left-hand, and right-hand motor activities are indicated as ‘0’, ‘1’, and ‘2’, respectively. The dataset comprises 11,524 samples, which are evenly distributed across the classes.

## Proposed methods

**Fig. 1:**
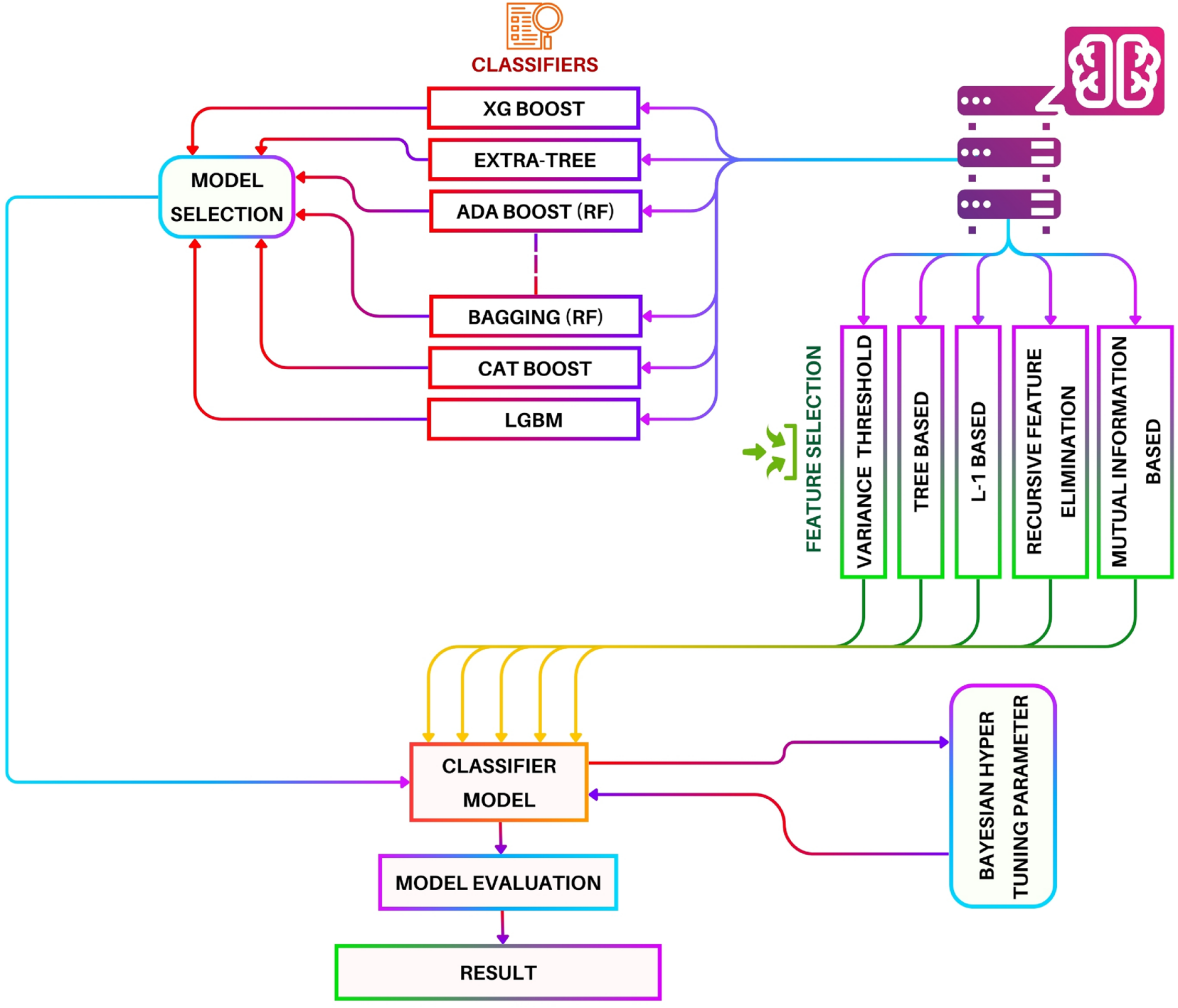
Overall methodology of the proposed machine learning model for motor imagery activity recognition.

In this paper, we exploit machine learning and deep learning models with feature selection and explanation AI methods to optimise EEG-based activity recognition by ensuring improved model accuracy and interpretability (see Fig. 1). This section introduces the methodology for detecting motor imagery activities. First, the dataset preprocessing will be described, which will be followed by the description of various machine learning models, feature selection methods, hyperparameter tuning, and the employed explainable models.

### Preprocessing

Since the raw EEG recordings are not publicly available, the preprocessing in this study starts from the released tabular feature files rather than from raw signal-level processing. The dataset contains EEG data of four subjects, with each subject’s data in a different CSV file. We merged the files into a single CSV file. It is a balanced dataset, hence it does not require a dataset balancing mechanism. Furthermore, there are no missing values. In addition, no encoding scheme is needed as the dataset contains no non-numeric feature values. To reduce the effect of outliers, we employ the standard scaler normalisation method, which centres data at 0 and scales it so that the standard deviation is 1. It is defined as:

1 $$\begin{aligned} x'=\frac{x-\mu }{\sigma } \end{aligned}$$,

where $\mu$ and $\sigma$ are the mean and standard deviation, respectively.

### Features selection methods

Feature selections are exploited in machine learning for several reasons, such as detection accuracy improvement, reduction of computational complexity, and so on^35^. In this paper, we apply five feature selection methods, namely the variance threshold method, extra tree-based method, mutual information, least absolute shrinkage and selection operator (LASSO) method, and RFE. These methods are implemented on the training set after splitting the dataset. Each model is trained using the selected features. Thereafter, the corresponding features are extracted from the test dataset, and the models are evaluated on the resulting dataset.

Variance threshold method: features with low variance can be regarded as constants and play a less important role in classification. For this reason, removing features with variance less than a threshold can significantly reduce the prediction time. In this method, before splitting the feature dataset, the variance of each feature is calculated^36^. Then, the features with variance lower than the threshold are removed.

Tree-based method: in tree-based feature selection, the importance of a feature depends on how well the feature splits the data for reducing impurity, such as the Gini index or entropy. The importance is quantified using the number of times the feature is used for splitting, the depth of the splits, and the reduction of impurity^37^. The feature importance of a feature *j* is defined as:

2 $$\begin{aligned} I_j = \sum _{t=1}^{T} \sum _{i \in t} \frac{N_s}{N} \, \Delta i(n) \cdot {\bf 1}\{\text {feature}(n) = j\} \end{aligned}$$,

where $N_s$ is the number of samples reaching the node *s*, *N* is the total number of samples, and ${\bf 1}\{\cdot \}$ is an indicator function, which equals 1 if the feature *j* is used to split the node *s*, and 0 otherwise.

When a node *s* is split using a feature, the Gini impurity reduction is calculated as^38^:

3 $$\begin{aligned} \Delta i(n) = i_{\text {Parent}} - \frac{N_{\text {left}}}{N_{\text {parent}}} \, i_{\text {left}} - \frac{N_{\text {right}}}{N_{\text {parent}}} \, i_{\text {right}} \end{aligned}$$,

where $i_{\text {Parent}}$ is the Gini impurity of the parent node, $i_{\text {left}}$ and $i_{\text {right}}$ are the Gini impurities of the left and right child nodes respectively, $N_{\text {parent}}$ is the number of samples in the parent node, and $N_{\text {left}}$ and $N_{\text {right}}$ are the number of samples going to the left and right child nodes, respectively. Tree-based feature selection method needs less testing time^39^.

Mutual information based: mutual information is a measure of how much information a random variable has about another random variable. This parameter can be used for feature selection by considering a feature and the target as two random variables. If a feature has more mutual information with respect to the target, we can regard that feature as highly relevant, thereby being an important feature for classification^40^. The measurement of mutual information between a feature value *x* and the target value *y* can be defined as:

4 $$\begin{aligned} I(X,Y) = \sum _{x \in X} \sum _{y \in Y} P(x,y) \log \left( \frac{P(x,y)}{P(x)P(y)} \right) \end{aligned}$$,

where *P*(*x*, *y*) is the joint probability between *x* and *y*, and *P*(*x*) and *P*(*y*) are the marginal probabilities of *x* and *y*, respectively. Similar to the tree-based method, this method can capture nonlinear relationships. However, as a univariate method, it does not consider interactions among features.

Recursive feature elimination: the recursive feature elimination is a sequential backward feature selection method that iteratively removes the least important features from the training dataset and tests the performance of the machine learning model against the test dataset^41^. This method starts with all features of the dataset and eliminates features iteratively, which finally ends up with a reduced set of the most important features. One of the limitations of this method is that it does not consider the correlation among the features. In addition, two weak features may not contribute significantly individually to the classification process; however, combined, they may have a good impact on classification^42^. Furthermore, RFE is a computationally expensive method due to its repeated training and testing. For this reason, this method is suitable for small datasets^43^.

L-1 feature selection: least absolute shrinkage and selection operator (LASSO), also known as L-1 feature selection, is a feature selection method that exploits L-1 regularization. It adds a penalty term to the loss function. The resulting LASSO estimates of the coefficient of a feature are given by:

5 $$\begin{aligned} \hat{\theta } = \arg \min _{\theta } \left\{ \frac{1}{2} \sum _{i}^{N} \left( y_i - \theta _0 - \sum _{j}^{p} x_{ij} \theta _j \right) ^2 + \mu \sum _{j}^{p} |\theta _j| \right\} \end{aligned}$$,

where *i* and *j* represent the indexes of the sample and feature, respectively, *x* and *y* represent the feature and target class, respectively, $\mu$ is the penalty parameter, and $\theta _j$ is the coefficient of feature *j*. The first term represents the loss, while the second term indicates the L-1 regularization penalty. While the first term tries to fit the data to the model, the second term tries to select the best set of features. A higher value of $\mu$ leads to an aggressive feature reduction, which makes the coefficients of more features equal to 0, thereby eliminating them from the feature set. On the other hand, $\mu =0$ reduces LASSO to the ordinary least-squares estimator, which keeps all features (i.e., no feature selection). The L-1 regularization method works well for large datasets. The downside of this method is that it may discard correlated features that are less effective individually but very effective when combined.

In this paper, these feature selection methods are applied with their stratified cross-validation over 5-folds to avoid overfitting, improve generalization, reduce bias, increase stability, and enhance the overall model’s performance, which is further discussed in the “Experimental results and analysis” section.

### Machine learning classifiers

In this paper, we employ 11 supervised machine-learning models to evaluate which model performs better for activity recognition. The ML models include both single classifiers such as logistic regression (LR)^44^, k-nearest neighbors^45^, decision tree^46^, and ensemble classifiers such as extra tree^47^, random forest^48^, bagging with LR as the base learner^49^, bagging with RF^50^, adaboost with RF as the base learner, cat boost^51^, light gradient boosting machine (LGBM)^52^, and extreme gradient boosting (XG-Boost)^53^. Deep learning models are not considered as the dataset is not large. Furthermore, gated recurrent unit (GRU) and LSTM have already been applied in^19^, and limited accuracy is obtained.

Logistic regression: logistic regression is one of the most commonly used functional supervised learning algorithms, particularly when dealing with binary classification tasks. It focuses on the probability of a specific outcome by applying a sigmoid activation function to a linear combination of input features, which are often used as input data nodes. The function helps determine whether a specific case is likely to occur or not. The boundaries of the function produce a value-saturating response, which places the most likely outcome between 0 and 1. Hence, it is widely preferred in a variety of applications where speed and timelines are important. Logistic regression is a lightweight, flexible, and computationally efficient model which is often used as an efficient solution for baseline of main starting tests.

K-nearest neighbors: k-nearest neighbors (KNN) is an instance-based and non-parametric machine learning algorithm. It is used for both classification and regression tasks. It finds the k nearest neighbors to a given input. For classification, it makes predictions based on the majority class, and for regression, it predicts based on the average value of those neighbor values. Besides having no pretraining, it employs the entire training data set and makes decisions at runtime. The strength of the KNN algorithm lies in its ability to handle different types of data and distance metrics, which directly affect its accuracy and efficiency. However, it is computationally expensive.

Decision tree: it is a tree-structured model that makes decisions based on feature values. It has a root node (decision points), branches (results or tests), and leaves (class labels). Each node tests a fine feature, which splits the data into subsets. The process repeats until a termination condition is met, such as maximum depth or minimum sample size. The algorithm maximizes Gini purity or minimizes entropy at each split. It handles both numerical and categorical data, making it versatile, but it is prone to overfitting. This model is easy to interpret and intuitive, but performance depends on the data’s complexity.

Extra tree: extra tree classifier is an ensemble learning algorithm that builds multiple decision trees with randomized split points. Using random feature selection at each split reduces variance and improves generalization compared to regular decision trees. It is particularly effective for high-dimensional data and resistant to overfitting. The algorithm works by splitting data based on random feature values, with the best split chosen based on the Gini impurity criterion. This makes it faster than traditional decision trees with less risk of overfitting.

Random forest: random forest classifier is another ensemble learning method that builds multiple decision trees. Each tree is trained on a random subset of the training data (bootstrap sampling). At each node, a random subset of features is considered for splitting. This diversification reduces variance and improves generalization. The final prediction is made through voting (classification) or averaging (regression). It is robust to noise and overfitting, making it popular in many real-world applications. It can handle high-dimensional data well and requires little pre-processing. Common parameters include the number of trees, depth, and the number of features to consider at each split.

Bagging (LR): bagging, with LR as the base classifier, is an ensemble technique that combines multiple logistic regression models. Each LR is trained on a different bootstrap sample of the original dataset. The final prediction is obtained through voting (for classification) or averaging (for probabilities). Bagging reduces variance by introducing diversification through random sub-sampling, which helps prevent overfitting. It is particularly effective with voting models such as LR that are sensitive to variances in the data. The technique is robust to noise and outliers, making it suitable for many real-world problems. Common parameters include the number of base estimators and the sample size for each bootstrap.

Bagging (RF): bagging can also be implemented by combining multiple decision trees, with each tree trained on a different bootstrap sample of the original data. The final prediction is obtained through majority voting (for classification) or averaging (for regression). Bagging with random forests introduces additional randomization by considering only a random subset of features at each split, further reducing variance and improving generalization. This makes it more robust to noise and less prone to overfitting than single tree models. The algorithm is effective in handling high-dimensional datasets and can help with feature selection. Common parameters include the number of trees, maximum depth, and the size of the feature subset.

AdaBoost (RF): adaboost (RF) classifier is an ensemble algorithm that combines multiple weak learners (usually decision trees) in a sequential manner. It assigns higher weights to incorrectly misclassified samples, focusing on the tough cases. Each weak learner is trained on a modified version of the training set, with more emphasis on previous mistakes. The final prediction is a weighted combination of all weak learners. AdaBoost with random forest accomplishes this by using bootstrapped samples and random feature selection, creating diversified models that contribute differently to the ensemble. This results in a robust model that is less sensitive to noise and overfitting. It is particularly effective for binary classification problems and can handle multiple classes.

CatBoost: this is an advanced gradient boosting algorithm designed for handling categorical features efficiently. It uses an ordered boosting, a sequential learning technique that prevents overfitting by using symmetrical binaries and ordered perturbations. The algorithm automatically handles categorical variables with the need for explicit encoding, reducing pre-processing effort. CatBoost implements a leaf-wise penalty to prevent overfitting and uses a Naïve prediction step scaling for better generalization. It is particularly effective on heterogeneous datasets and can handle missing values natively. The algorithm includes built-in processing features and a parameter for controlling the speed of learning. CatBoost is known for its robustness and ease of use.

Light gradient boosting machine (LGBM): LGBM classifier is an efficient gradient boosting algorithm that uses a leaf-wise tree growing strategy. By focusing on distribution-based sampling, it reduces memory usage and accelerates training time while maintaining accuracy. It is ideal for large-scale datasets and can handle multiple classes effectively. The algorithm implements histogram-based binning for continuous and optimized leaf-wise splits. LGBM also includes built-in parallelization and GPU support, enabling fast training on multi-core CPUs. The model is robust to overfitting and performs well on balanced and imbalanced datasets.

Extreme gradient-boost (XG-Boost): it is a very popular gradient boosting algorithm that combines tree-based learning with linear modeling. It uses a combination of regularization techniques (L1 and L2) and grows trees layer-by-layer, by minimizing a loss function while adding complexity penalties. The algorithm implements parallelized training, distribution-based computation, and efficient handling of missing values. XG-Boost is known for its speed and performance, particularly on large datasets. It also supports custom loss functions and evaluation metrics, making it versatile for both classification and regression problems. The model is robust to overfitting and can handle high-dimensional data.

**Algorithm 1:**
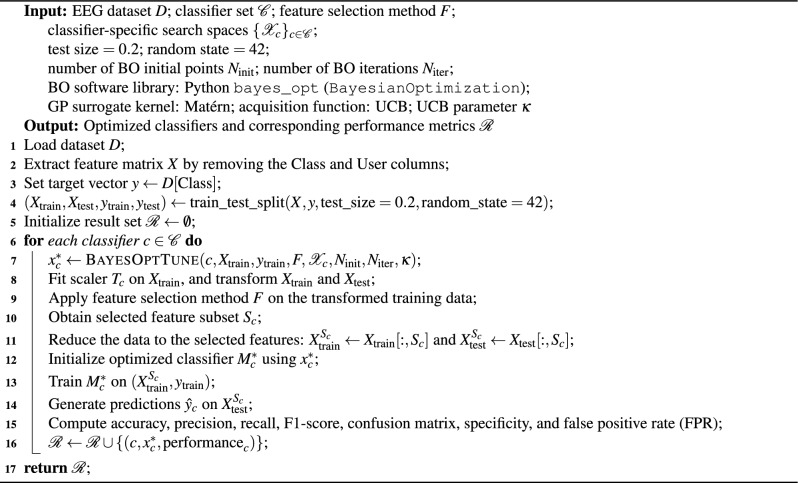
Overall classification pipeline with Bayesian optimization.

**Algorithm 2:**
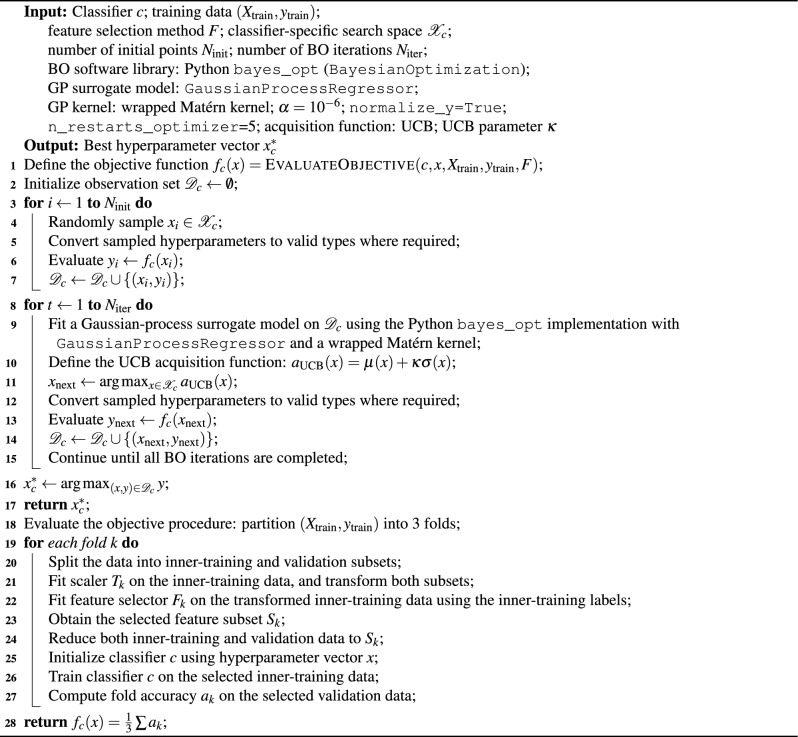
BayesOptTune: Bayesian optimization for classifier-specific hyperparameter tuning.

### Model optimization

Hyperparameter tuning enhances model performance by identifying the best values for learning parameters (e.g., learning rate, regularization level, tree count) that are not directly learned from the data. Optimal tuning can improve accuracy, prevent overfitting or underfitting, and improve generalization abilities to unseen data^54^. Prominent hyperparameter tuning techniques include the following:

Grid search: systematically investigates all possible combinations within a predefined range of values. It is guaranteed to detect the best combinations within that grid and is simple to implement. However, it is computationally expensive when the number of hyperparameters or possible values is large.

Random search: it tests randomly generated parameter conditions, statistically more efficient than exhaustive search. It is faster than grid search, but no guarantee of finding the absolute best combinations and results may differ depending on random sampling.

Optimization algorithms: optimization-based hyperparameter tuning uses systematic search strategies to find the best hyperparameter values for a model. Methods such as Bayesian optimization, genetic algorithms, and gradient-based approaches leverage previous evaluations to efficiently explore the search space. It is more efficient and can converge to high-performance configurations quicker than the brute-force method. In this paper, we use Bayesian optimization for hyperparameter tuning of the classifiers employed in detecting MI activity. Next, we describe the working principle of this algorithm for hyperparameter tuning.

The Bayesian method constructs a probabilistic surrogate model (typically a Gaussian process) to approximate the objective function. It then uses this model to balance exploration of uncertain areas with exploitation of promising results, making it ideal for complex model tuning in high-dimensional spaces. The overall classification workflow is given in Algorithm 1, whereas the Bayesian optimization (BO) procedure used within that workflow is detailed in Algorithm 2, and each of its steps is described below:

**Fig. 2:**
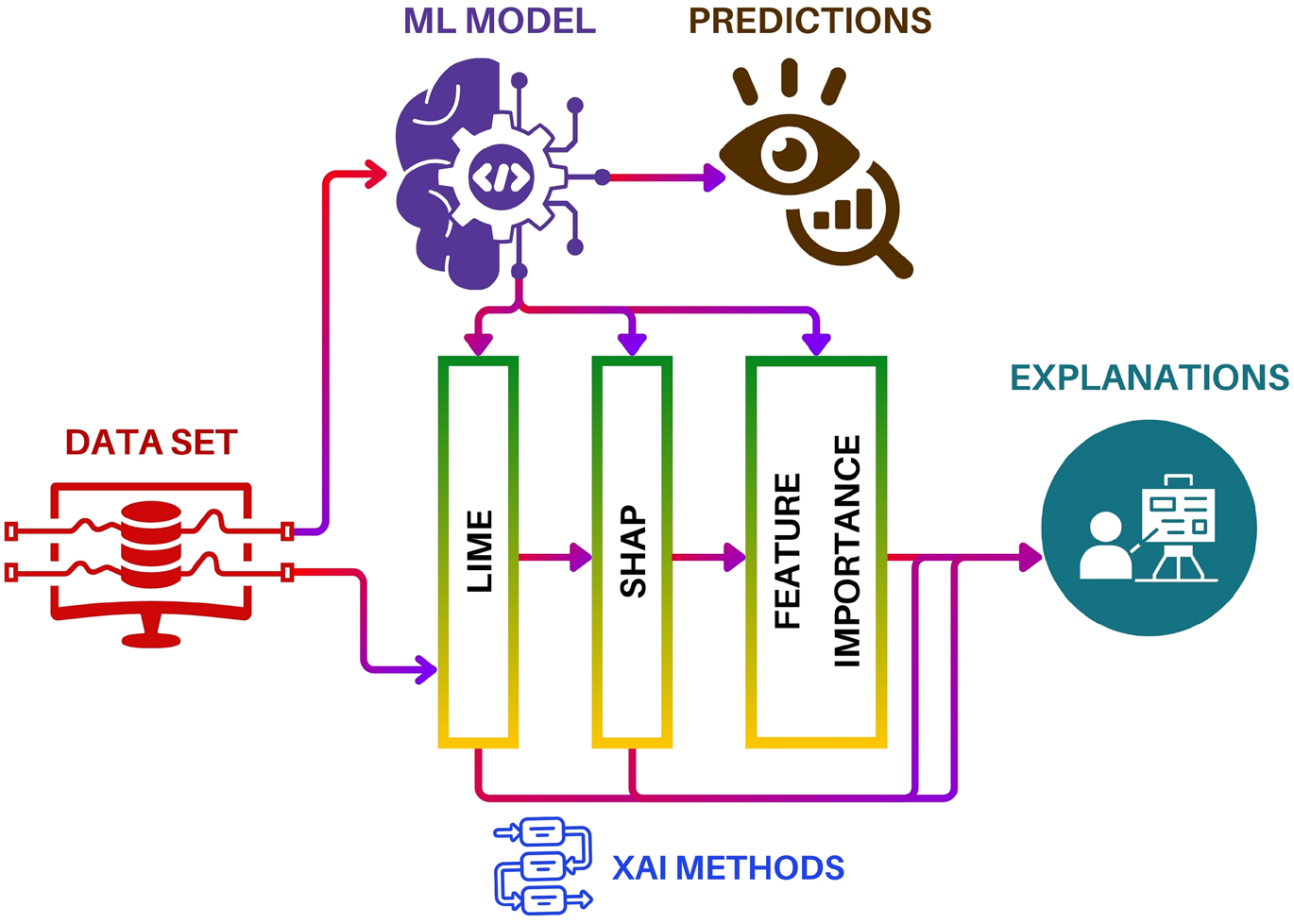
Architecture of the explainability analysis.

To determine the optimal hyperparameter configuration for each classifier, the BO objective was defined as the mean 3-fold cross-validation accuracy computed only on the training data. A Gaussian process (GP) surrogate model was employed to approximate the objective function, generating a predictive mean $\mu (x)$ and predictive standard deviation $\sigma (x)$ for a target hyperparameter vector *x*. The subsequent hyperparameter configuration was selected using the upper confidence bound (UCB) acquisition function, which balances exploitation of promising regions and exploration of uncertain regions during the search process^55–57^. For the unconstrained optimization setting used here, the library employs UCB as the default acquisition function and internally uses a GaussianProcessRegressor with a wrapped Matérn kernel $(\nu = 2.5)$, together with $\alpha = 10^{-6}$, normalize_y = True, and n_restarts_optimizer = 5^58^.

Algorithm 1 describes the overall classification scheme. In lines 1–4, the EEG dataset is loaded and processed, the *Class* label is assigned as the target vector, and the dataset is divided into training and testing subsets using an 80:20 split with a fixed random state. Line 5 initializes the result set *R* to store the optimized parameters for all classifiers. In lines 6–16, the algorithm iterates through each classifier in the candidate set. Line 7 invokes Algorithm 2 to obtain the best hyperparameter vector $x_c^*$ from the classifier-specific search space $X_c$. In lines 8–11, the scaler and feature selector are fitted only on the training data, and the selected feature subset $S_c$ is obtained. In line 12, the optimized classifier $M_c^*$ is initialized using the returned hyperparameter vector $x_c^*$. In lines 13 and 14, the optimized classifier is trained on the selected training data and then used to generate predictions on the selected test data. Line 15 computes the evaluation metrics, and line 16 stores the classifier, its optimal hyperparameter vector, and its corresponding performance measures in the result set *R*. Finally, line 17 returns the complete set of optimized results.

Algorithm 2 describes the Bayesian optimization pipeline used in line 7 of Algorithm 1. In line 1, the objective function $f_c(x)$ is defined through the cross-validation evaluation procedure *EvaluateObjective*. Line 2 initializes the observation set $D_c$ to store the evaluated hyperparameter configurations and their corresponding objective values. In lines 3–7, an initial exploration phase is performed by randomly sampling $N_{\textrm{init}}$ hyperparameter vectors from the classifier-specific search space $X_c$, converting them to valid parameter types where required, evaluating the objective function, and storing the results in $D_c$. In lines 8–15, the sequential BO phase is executed. At each iteration, line 9 fits a GP surrogate model on the accumulated observations, lines 10–11 define and maximize the UCB acquisition function to find the next target hyperparameter vector $x_{\textrm{next}}$, lines 12 and 13 convert and evaluate the sampled parameters, and line 14 appends the new findings to $D_c$. Line 16 identifies the best hyperparameter vector $x_c^*$ from the BO iterations and line 17 returns it to Algorithm 1.

In the fold-wise evaluation routine of lines 18–28 of Algorithm 2, the training data are randomly separated into three folds, and each fold is processed by fitting the scaler and feature selector only on the inner-training subset, training classifier *c* with hyperparameter vector *x*, and computing the validation accuracy. The final objective value returned to the BO routine is the mean of the three-fold accuracies.

In this study, BO was adopted as a standard optimization strategy that iteratively updates a surrogate model of the objective function and uses an acquisition rule to select the next point for evaluation^55,56^. The use of a GP surrogate is also standard, with the posterior predictive mean $\mu (x)$ and predictive standard deviation $\sigma (x)$ providing the quantities required by the UCB rule^57^. The classifier-specific search spaces were defined according to the tunable parameters of each classifier, such as the number of estimators, maximum tree depth, learning rate, minimum samples per split, minimum samples per leaf, feature subsampling ratio, and other classifier-dependent parameters.

**Table 1:** Performance [%] of single classifiers with all features and default hyperparameters.

Classifiers	Accuracy	Recall	FPR	Specificity	FNR	Precision	F1-score
Logistic regression	45.09	45.40	27.30	72.43	54.40	44.66	44.90
K-neighbors	68.53	72.50	13.67	86.23	27.40	68.66	70.26
Decision tree	59.55	56.53	21.33	78.27	43.37	58.67	57.57

**Table 2:** Performance [%] of ensemble classifiers with all features and default hyperparameters.

Classifiers	Accuracy	Recall	FPR	Specificity	FNR	Precision	F1-score
Extra tree	86.80	83.37	8.30	91.70	16.60	84.33	83.80
Random forest	83.37	81.64	9.17	90.82	18.33	82.67	82.13
Bagging (LR)	41.10	41.57	29.23	70.77	55.40	44.00	42.67
Bagging (RF)	82.20	81.03	9.37	90.60	18.83	81.67	81.37
AdaBoost (RF)	82.25	82.20	8.82	91.10	17.70	82.00	82.33
CatBoost	82.03	80.83	9.54	90.40	19.17	81.67	81.23
LGBM	76.35	74.97	12.47	87.50	25.00	80.00	77.40
XG-Boost	81.77	81.97	9.10	90.83	18.23	80.33	81.13

### Explainable AI

Explainable machine learning refers to techniques and methods that enable humans to understand, translate, and trust the decisions made by machine learning algorithms. It breaks down the black-box nature of complex models, providing insights into how and why predictions are made. It allows humans to understand the model’s decision-making process, enabling better decision-making. It identifies problems in the model or data, improving performance and reliability. It is transforming the way we interact with machine learning models, making them more accessible, fair, and responsible. The proposed architecture of the explainable AI is shown in Fig. 2. In what follows, two popular explainable machine learning methods will be described.

LIME: local interpretable model-agnostic explanations (LIME) is a local approach to explain the predictions of any classifier by approximating it locally with an interpretable model. The method was proposed in^59^ to enable explanations in machine learning, even when the base model is a black-box algorithm. It works by generating synthetic data points around the instance to be explained and using these to build a linear model that best approximates the original model’s behavior in that local region. LIME generates samples by randomly perturbing with Gibbon’s probabilities, emphasizing local proximity. It uses a simple linear model that minimizes the loss function while preserving local fidelity, typically defined as^59^:

6 $$\begin{aligned} L(f, g, \pi _x) = \sum _{z \in Z} \pi _x(z) \, \big (f(z) - g(z)\big )^2 \end{aligned}$$,

where *f* is the complex model, *g* is the simple model, $\pi _x(z)$ is a weight based on proximity to the input *x*, and *Z* is the set of perturbed samples. This approximates the behavior of the complex model locally, providing feature importance scores to highlight which inputs most influence the decision. LIME’s strength lies in its versatility and model-agnostic nature, making it a widely used tool in XAI.

SHAP: SHapley Additive exPlanations (SHAP) is a formal framework for explaining the output of any machine learning model, based on concepts from game theory and sophisticated linear explanations. Centered around the idea of “SHAP values”, it provides a unified approach to assigning importance values to each feature for a given prediction that reflects its contribution to the difference between the model’s actual output and its expected output. This is achieved through a mathematically robust formalism that guarantees feature contributions based on all possible feature combinations. The SHAP value for the feature *i* is given by^60^:

7 $$\begin{aligned} \phi _i = \sum _{S \subseteq F \setminus \{i\}} \frac{|S|!\,\big (|F| - |S| - 1\big )!}{|F|!} \,\big [ f(S \cup \{i\}) - f(S) \big ] \end{aligned}$$,

where *S* is a subset of features that does not include *i*, *f*(*S*) is the model’s prediction using only the features in subset *S*, |*S*| is the number of features in subset *S*, and |*F*| is the total number of features. The technique was proposed by Scott M. Lundberg and Su-In Lee in 2017^61^. SHAP has emerged as one of the most robust and theoretically grounded methods for explaining model decisions. Its ability to provide hard assertion contributions while maintaining theoretical consistency makes it a powerful XAI tool.

**Table 3:** Bayesian optimized hyperparameters for the ML models.

ML models	Parameters	Choices	Selected
LR	c	(1, 20)	8.0865
	n_iteration	(100, 1000)	843
	tol	(0.001, 1)	0.0015773
KNN	n_neighbors	(1, 20)	1
	weight	{uniform, distance}	uniform
	p	(1, 15)	1.0016
DT	n_estimators	(3, 20)	18
	min_samples_split	(2, 50)	2
	min_samples_leaf	(1, 20)	1
	max_features	(0.1, 0.9)	0.7559
ET	n_estimators	(50, 500)	421
	min_samples_split	(2, 20)	3
	min_samples_leaf	(1, 10)	1
	max_features	(0.1, 0.9)	0.9
RF	n_estimators	(100, 600)	600
	min_samples_split	(2, 10)	2
	min_samples_leaf	(1, 10)	1
	max_features	(0.1, 0.5)	0.3189
	max_depth	(10, 50)	37
Bagging (LR)	bag_n_estimators	(5, 20)	14
	bag_max_samples	(0.5, 1.0)	0.6942
	lr_C	(0.001, 100.0)	59.8690
	lr_solver	(0, 1)	lbfgs
	lr_max_iter	(100, 1000)	820
Bagging (RF)	bag_n_estimators	(5, 20)	19
	rf_n_estimators	(50, 150)	96
AdaBoost (RF)	adaboost_n_estimators	(10, 50)	39
	learning_rate	(0.01, 1.0)	1
	rf_n_estimators	(50, 150)	57
	rf_max_depth	(5, 15)	11
	rf_max_features	(0.05, 0.2)	0.05
CatBoost	iterations	(100, 1000)	970
	learning_rate	(0.01, 0.3)	0.2362
	depth	(4, 10)	7
	l2_leaf_reg	(1, 10)	7.2580
	subsample	(0.5, 1.0)	0.9978
LGBM	num_leaves	(20, 300)	253
	max_depth	(3, 12)	9
	learning_rate	(0.001, 0.3)	0.095
	n_estimators	(50, 500)	486
	min_child_samples	(5, 100)	6
	subsample	(0.1, 1.0)	0.2911
	colsample_bytree	(0.5, 1.0)	0.9331
XG-Boost	n_estimators	(100, 500)	307
	max_depth	(3, 10)	8
	learning_rate	(0.01, 0.3)	0.3
	min_child_weight	(1, 10)	4.0783
	subsample	(0.6, 1.0)	1
	colsample_bytree	(0.6, 1.0)	1
	gamma	(0, 0.5)	0

Feature importance: feature importance is a fundamental concept in XAI that measures the contribution of each input variable to a model’s prediction accuracy. It is widely used in machine learning to understand the relationship between features and target variables, and to identify the most critical features that influence the model’s decisions. Feature importance methods can be calculated using various techniques, including permutation importance, Gini impurity, and mean decrease in accuracy. These methods are essential for building trustworthy models, ensuring their decisions are transparent and interpretable.

Gini impurity methods are among the most commonly used techniques for calculating feature importance. It measures the frequency of a feature being used to split the data and its contribution to reducing the Gini impurity. The Gini impurity for a feature *f* can be expressed as^38^:

8 $$\begin{aligned} I(f) = \sum _{t \in T} \sum _{n \in N_t(f)} \Delta I_n \end{aligned}$$,

where *I*(*f*) is the importance of feature *f*, *T* is the set of all trees in the ensemble, $N_t(f)$ is the set of nodes in tree *t* where feature *f* is used for splitting, and $\Delta I_n$ is the reduction in impurity at node *n*, defined as^39^:

9 $$\begin{aligned} \Delta I_n = I_{\text {parent}} - \big ( w_{\text {left}} \cdot I_{\text {left}} + w_{\text {right}} \cdot I_{\text {right}} \big ) \end{aligned}$$,

where $I_{\text {parent}}$ is the impurity of the parent node, $I_{\text {left}}$ and $I_{\text {right}}$ are the impurities of the left and right child nodes, respectively, and $w_{\text {left}}$ and $w_{\text {right}}$ are the proportions of samples going to the left and right children, respectively.

## Experimental results and analysis

In this section, we will evaluate the performance of the proposed machine learning framework for activity detection. We will consider three different scenarios: detection performance with (a) all features and default hyperparameters, (b) selected features and default hyperparameters, and (c) selected features and optimized hyperparameters. Using this evaluation framework, we will be able to shed light on the most contributory factor in improved activity recognition. Thereafter, XAI will be employed to explain the reasoning behind the detection.

**Table 4:** Performance [%] of single classifiers with all features and optimized hyperparameters.

Classifiers	Accuracy	Recall	FPR	Specificity	FNR	Precision	F1-score
Logistic Regression	46.01	45.40	27.30	72.43	54.40	46.00	45.60
K-Neighbors	86.89	87.03	6.53	93.43	14.07	87.00	86.97
Decision Tree	60.32	60.17	20.87	79.37	43.37	60.33	60.20

**Table 5:** Performance [%] of ensemble classifiers with all features and optimized hyperparameters.

Classifiers	Accuracy	Recall	FPR	Specificity	FNR	Precision	F1-score
Extra tree	89.54	89.43	7.23	94.37	13.93	90.00	89.70
Random forest	85.89	85.83	9.17	90.82	18.33	86.00	85.87
Bagging (LR)	45.83	45.77	28.90	70.77	55.40	45.67	45.67
Bagging (RF)	82.24	81.43	9.37	90.60	18.83	82.33	81.70
AdaBoost (RF)	86.28	86.77	2.73	96.73	16.50	86.00	86.36
CatBoost	85.02	84.83	9.54	90.40	19.17	85.00	84.90
LGBM	88.15	87.80	6.03	93.93	13.67	88.00	87.87
XG-Boost	80.77	81.97	9.10	90.83	18.23	80.67	81.30

**Table 6:** Accuracy [%] of ensemble classifiers with the selected features and default hyperparameters.

Feature selection methods	ET	RF	Bagging (LR)	Bagging (RF)	AdaBoost (RF)	CatBoost	LGBM	XG-Boost
Variance threshold	87.37	85.59	43.14	83.42	85.72	81.29	79.38	80.73
Extra tree-based	90.28	86.94	44.53	85.59	85.81	83.42	80.51	82.47
Recursive feature elimination	90.54	86.37	44.18	85.55	86.11	82.94	80.34	82.73
Mutual information based	87.15	84.81	43.97	83.38	82.25	83.59	80.90	80.86
L-1	85.63	82.73	41.54	81.42	82.34	79.04	76.52	78.43

**Table 7:** Accuracy [%] of ensemble classifiers with the selected features and optimized hyperparameters.

Feature selection methods	No. of selected features	ET	RF	Bagging (LR)	Bagging (RF)	AdaBoost (RF)	CatBoost	LGBM	XG-Boost
Variance threshold	52	90.62	86.72	42.62	83.68	85.98	84.59	87.54	84.42
Extra tree-based	53	90.58	87.63	44.40	85.81	88.28	85.55	88.80	85.94
Recursive feature elimination	50	91.88	88.30	44.05	86.11	88.76	85.42	90.06	87.11
Mutual information based	65	90.10	86.50	45.23	83.77	87.20	85.42	87.50	85.07
L-1	77	90.19	86.28	41.15	83.38	86.46	84.94	88.24	84.72

**Fig. 3:**
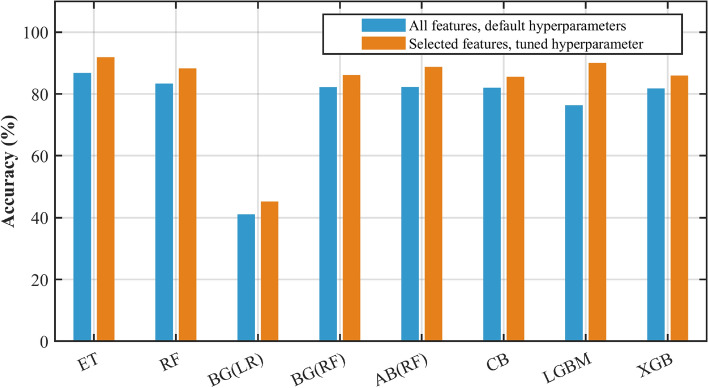
Impact of the feature selection and hyperparameter tuning on the classifiers performance.

### Evaluation metrics

In multi-class classification, each instance belongs to one of $K$ mutually exclusive classes. Evaluating such models requires extending traditional binary classification metrics. In this work, we adopt the *macro-averaging* approach, where each metric is computed independently for each class using a one-vs-rest strategy, and then averaged equally across all classes. Let $TP_i$, $FP_i$, $FN_i$, and $TN_i$ denote the number of true positives, false positives, false negatives, and true negatives for class $i$, respectively.

#### Accuracy

Accuracy measures the overall correctness of the classifier, indicating the proportion of total predictions that are correct, regardless of class^62^:

10 $$\begin{aligned} \text {Accuracy} = \frac{\sum _{i=1}^{K} c_{ii}}{\sum _{j=1}^{K} \sum _{k=1}^{K} c_{jk}} \end{aligned}$$

Here, $c_{ii}$ represents the number of correctly classified instances of class $i$, *K* represents the number of classes, the denominator is the total number of instances.

#### Precision

Precision evaluates the classifier’s ability to avoid false positives. It quantifies how many of the predicted instances for each class are actually correct. High precision indicates that the classifier rarely mislabels instances from other classes as class $i$. It is defined as:

11 $$\begin{aligned} \text {Precision} = \frac{1}{K} \sum _{i=1}^{K} \frac{TP_i}{TP_i + FP_i} \end{aligned}$$

#### Recall

Recall, also called true positive rate (TPR), assesses the classifier’s ability to correctly identify all relevant instances of a class. It measures the proportion of actual instances of class $i$ that are correctly predicted:

12 $$\begin{aligned} \text {Recall} = \frac{1}{K} \sum _{i=1}^{K} \frac{TP_i}{TP_i + FN_i} \end{aligned}$$

High recall implies that the model is effective at retrieving instances from each class without missing many.

**Fig. 4:**
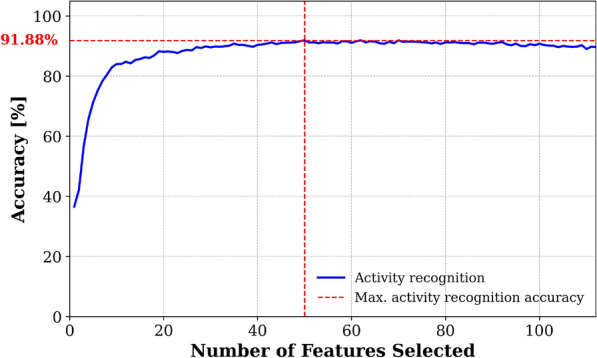
Impact of the number of features on the accuracy of extra tree with RFE.

#### False Positive Rate (FPR)

This metric quantifies the rate at which the classifier incorrectly labels instances from other classes as class $i$. Lower values are desirable. It is a measure of over-prediction for each class:

13 $$\begin{aligned} \text {FPR} = \frac{1}{K} \sum _{i=1}^{K} \frac{FP_i}{FP_i + TN_i} \end{aligned}$$

#### Specificity

Also known as true negative rate (TNR), this metric measures the proportion of actual negatives correctly identified. It reflects the classifier’s ability to correctly reject instances that do not belong to class $i$:

14 $$\begin{aligned} \text {TNR} = \frac{1}{K} \sum _{i=1}^{K} \frac{TN_i}{TN_i + FP_i} \end{aligned}$$

High specificity means the classifier is conservative in its predictions and avoids false positives.

#### False negative rate (FNR)

This rate captures how often actual instances of class $i$ are missed by the classifier. Lower FNR values are critical in applications where missing positive cases is costly such as intrusion detection. It is defined as:

15 $$\begin{aligned} \text {FNR} = \frac{1}{K} \sum _{i=1}^{K} \frac{FN_i}{TP_i + FN_i} \end{aligned}$$

#### F1 score

The F1 score balances precision and recall through its harmonic mean:

16 $$\begin{aligned} \text {F1 Score} = \frac{1}{K} \sum _{i=1}^{K} \left( 2 \times \frac{TP_i}{2TP_i + FP_i + FN_i} \right) \end{aligned}$$

A high F1 score indicates both high precision and high recall, making it a robust metric for general performance assessment.

**Table 8:** Comparison with the existing work [%].

Paper	Dataset	Accuracy	Precision	Recall	F1-score
GRU^19^	EEG-HM	70.41	–	–	–
LSTM^19^	EEG-HM	69.9	–	–	–
ADBN+FNO^63^	PhysioNet	94.10	93.60	94.00	93.80
GPFMTL^64^	PhysioNet	80.99	–	–	8.01
SVM-SA^65^	PhysioNet	86.47	–	78.00	83.00
LSTM^66^	PhysioNet	91.25	–	83.33	–
SqueezeNet^66^	PhysioNet	60.60	–	83.33	–
NN^67^	PhysioNet	92.90	–	–	–
Proposed	EEG-HM	**91.88**	**91.40**	**91.88**	**91.70**
Proposed	PhysioNet	**98.77**	**98.77**	**98.77**	**98.77**

Significant values are in bold.

**Table 9:** Performance comparison of DL baselines [%].

DL models	Accuracy	Precision	Recall	F1-score
1D-CNN	77.60	77.75	77.63	77.62
Residual-MLP	77.47	78.01	77.46	77.49
BiLSTM	70.14	70.18	70.13	70.13
**Proposed**	**91.88**	**91.40**	** 91.88**	**91.70**

Significant values are in bold.

**Table 10:** Performance of classifiers using autoencoder-derived features [%].

Model	Accuracy	Recall	FPR	Specificity	FNR	Precision	F1-score
KNN	80.73	80.70	9.63	90.37	19.30	80.75	80.71
Extra tree	79.60	79.56	10.20	89.80	20.44	79.59	79.56
LightGBM	77.73	77.72	11.13	88.88	22.28	77.73	77.72
Random forest	76.26	76.24	11.86	88.14	23.76	76.27	76.24
XGBoost	75.78	75.75	12.10	87.90	24.25	75.78	75.76
AdaBoost RF	75.52	75.52	12.23	87.77	24.48	75.85	75.55
Bagging RF	75.35	75.32	12.32	87.68	24.68	75.33	75.32
CatBoost	74.48	74.46	12.75	87.25	25.54	74.50	74.47
Logistic regression	65.93	65.88	17.03	82.97	34.12	65.92	65.90
Bagging LR	65.84	65.82	17.07	82.93	34.18	65.88	65.84
Decision tree	48.57	49.01	25.47	74.53	50.99	60.69	46.06

**Fig. 5:**
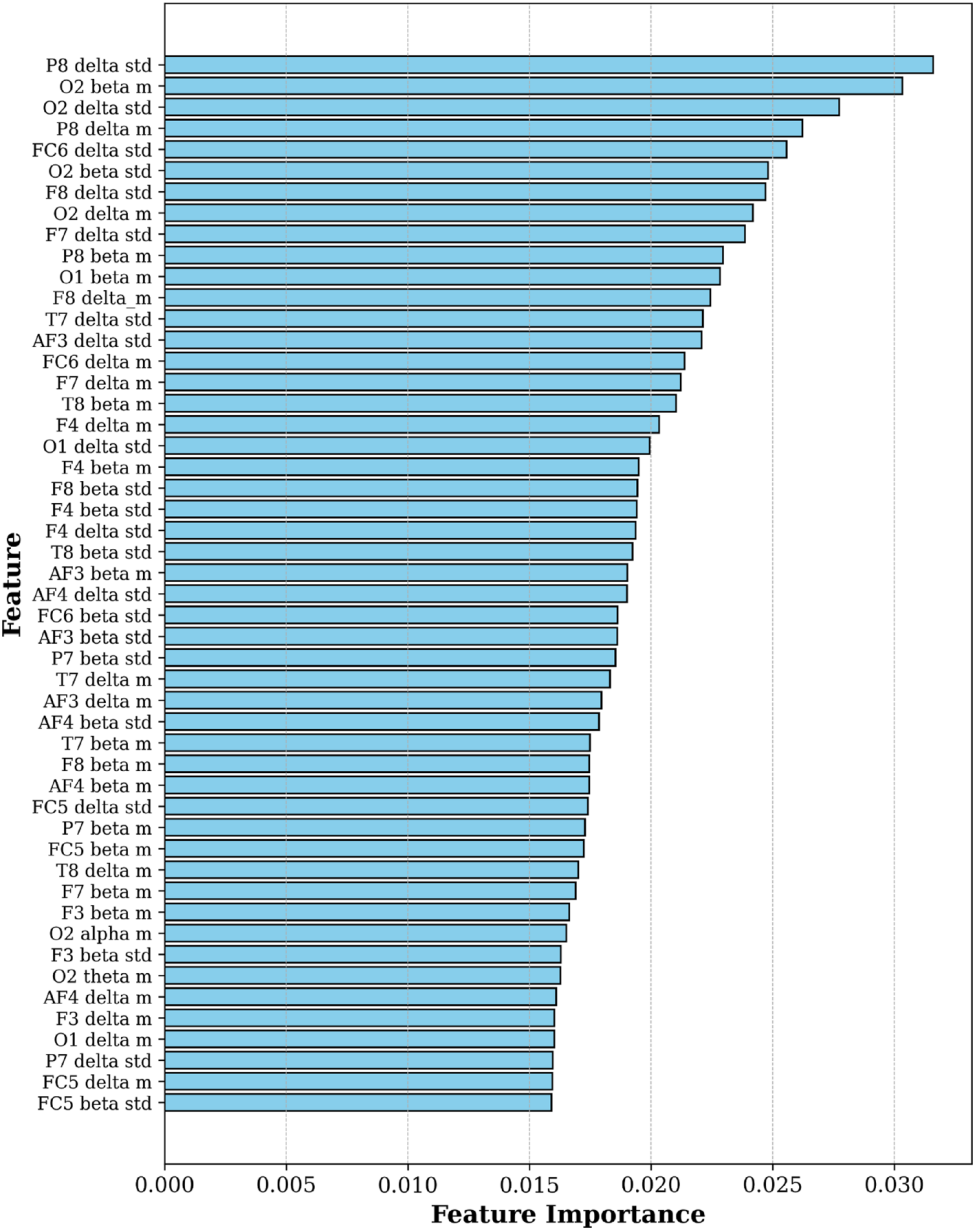
Feature importance of the 50 most influential features selected by RFE.

**Fig. 6:**
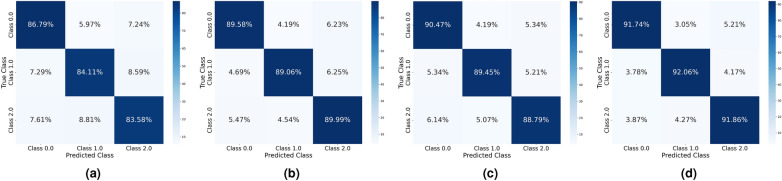
Confusion matrices for the Extra Tree classifier. (**a**) Default hyperparameters, all features, (**b**) hyperparameter tuning, all features, (**c**) default hyperparameters, RFE, (**d**) hyperparameter tuning with RFE.

**Fig. 7:**
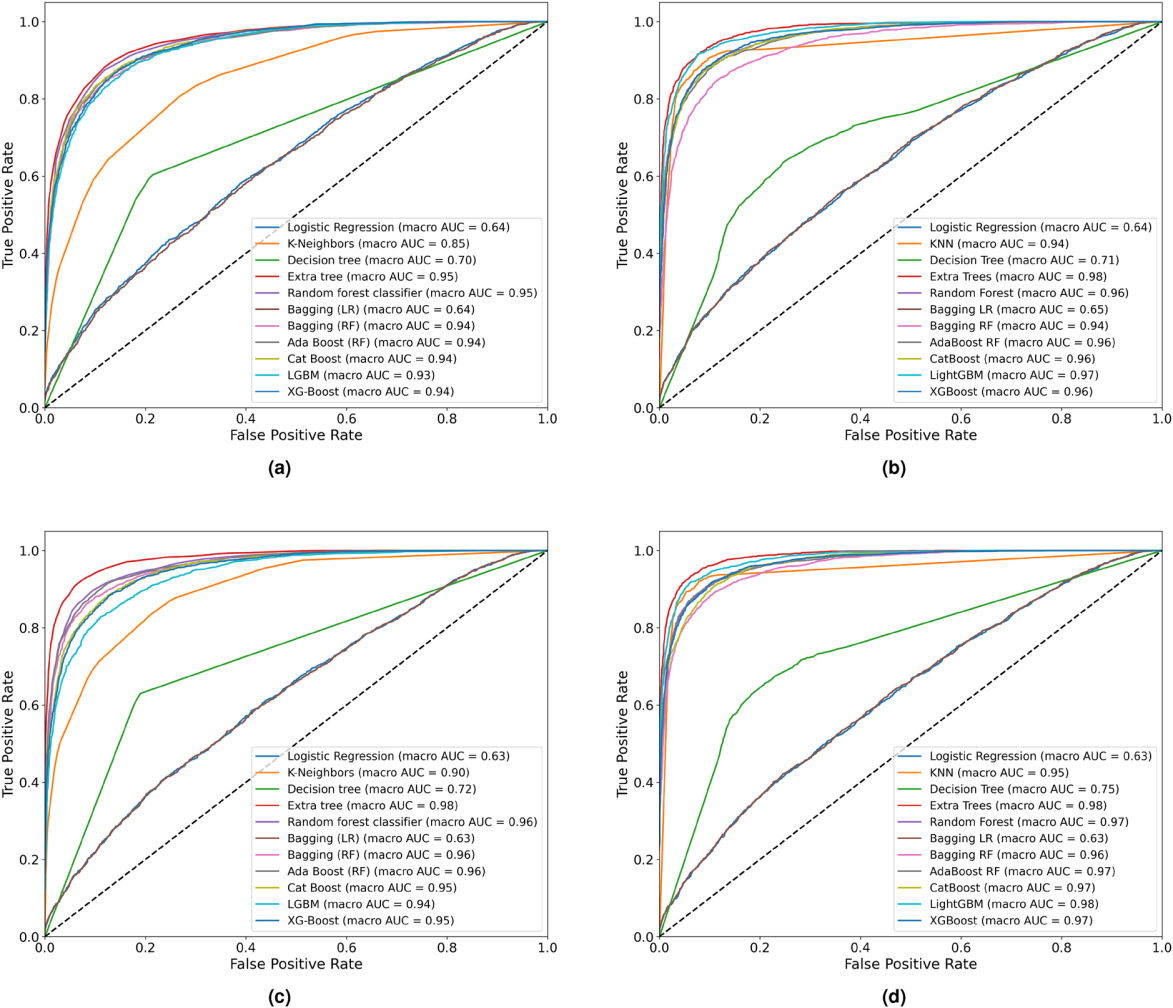
ROC of the classifiers over all classes. (**a**) Default hyperparameters, all features, (**b**) hyperparameter tuning (HPT), all features, (**c**) default hyperparameters, RFE-selected features, (**d**) HPT with RFE.

### Results and analysis

There are many machine learning classifiers that can be employed for classification. Among them, initially, we will apply both single and ensemble classifiers to find the better-performing classifiers. Then, further processing will be done with the selected classifiers. Table 1 shows the performance of the most commonly used three single classifiers: logistic regression (LR), k-nearest neighbors (KNN), and a decision tree. While the KNN provides the highest accuracy of 68.53%, the worst performance is observed from LR, which achieves 45.09% accuracy. Table 2 shows the performance of various ensemble classifiers in the same scenario: all features and default hyperparameters. Except for the bagging classifier with the logistic regression as the base classifier, all ensemble models outperform the single classifiers by a large margin. The extra tree classifier is found to be the best ensemble model by attaining 86.8% of accuracy, while the random forest achieves the second-highest accuracy of 83.37%. The bagging (RF), adaboost (RF), catboost, and XGboost exhibit nearly identical performance, with accuracies ranging from 81.7% to 82.25%. It is also revealed that the precision is lower than the accuracy for all classifiers except LGBM. This stems from the higher TNR and FNR compared to the recall and specificity. Interestingly, LGBM is the only classifier whose precision is notably higher than its accuracy. On the other hand, it has a much lower recall compared to other models. This is due to the higher FNR. Another interesting observation is that the specificity is always higher than recall. This can be explained as follows: when the model detects a specific class in a multi-class setting, all other classes are considered as negatives for that class. Even in a balanced dataset, the number of negative samples for any single class is higher than the number of positive samples for that class because negatives consist of all other classes combined. This larger negative set increases the probability of correct classification for negatives, leading to higher specificity.

**Fig. 8:**
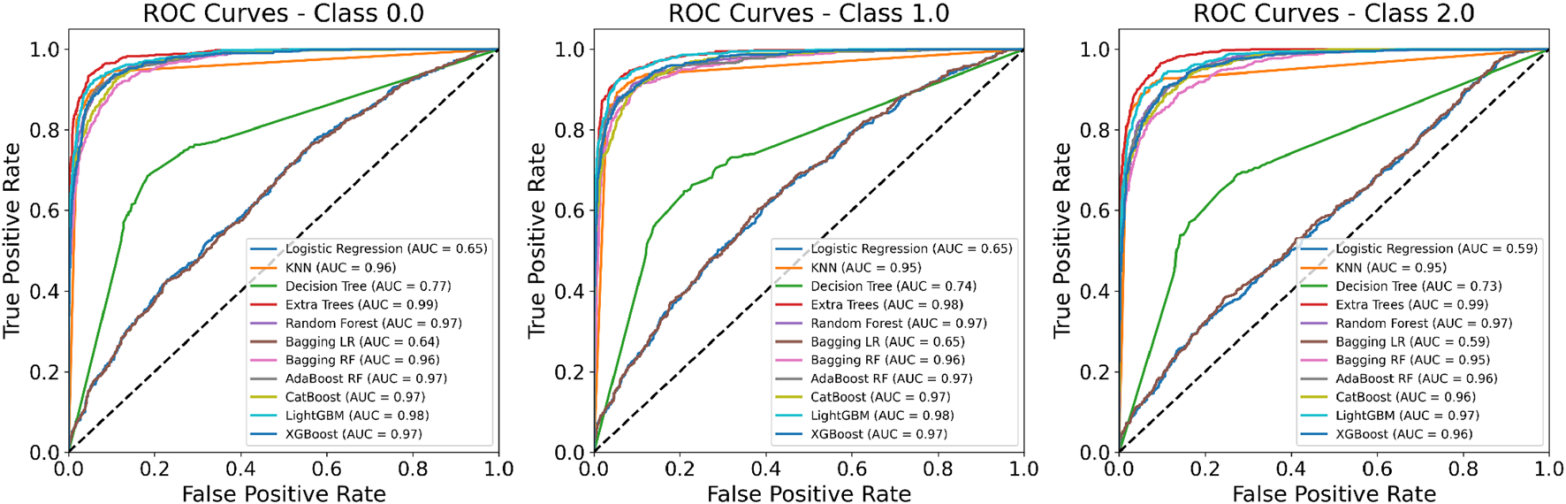
ROC of the all-optimized models with RFE feature selection for individual classes.

**Table 11:** Extra tree with RFE feature selection inside 5-fold cross-validation [%].

Fold	Accuracy	Recall	Precision	F1-score	Specificity	FPR
1	91.41	91.41	91.41	91.41	95.70	4.30
2	91.97	91.97	91.98	91.96	95.99	4.01
3	91.84	91.84	91.84	91.84	95.92	4.08
4	91.10	91.10	91.11	91.10	95.55	4.45
5	90.67	90.67	90.70	90.67	95.33	4.67

**Fig. 9:**
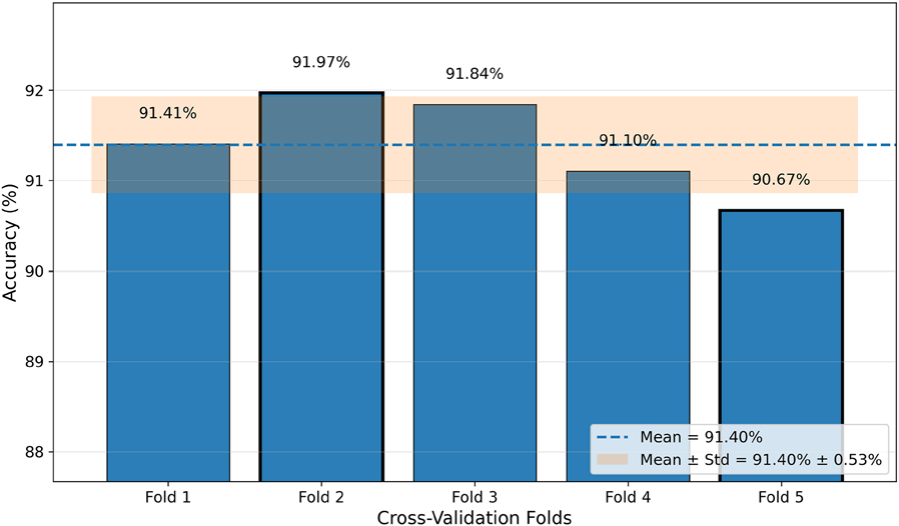
Fold-wise accuracy of the Extra Tree classifier with RFE under leakage-free 5-fold cross-validation.

**Table 12:** Descriptive statistics of the evaluated models across repeated experiments [%].

Model	Mean acc.	Std. acc.	CI95 low	CI95 high	Avg. train time (s)	Avg. test time (s)	Folds
Extra tree	91.70	0.56	91.55	91.86	11.941258	0.146997	50
LightGBM	89.70	0.59	89.54	89.87	12.008645	0.175840	50
KNN	89.60	0.53	89.45	89.74	0.002002	0.634921	50
Random forest	88.31	0.71	88.11	88.50	52.240374	0.202912	50
AdaBoost RF	87.78	0.72	87.58	87.99	22.478308	0.924704	50
XGBoost	87.33	0.73	87.13	87.53	5.590904	0.030218	50
CatBoost	86.88	0.73	86.68	87.08	33.074009	0.042616	50
Bagging RF	86.79	0.67	86.60	86.97	45.977631	0.718182	50
Decision tree	61.89	1.51	61.47	62.30	0.820907	0.000891	50
Logistic regression	43.60	1.00	43.32	43.87	0.847490	0.000730	50
Bagging LR	43.59	0.95	43.33	43.86	248.022531	0.125090	50

**Table 13:** Friedman test results for overall performance differences among the evaluated models.

Chi2	p-value
479.134510	1.26486e-96

**Table 14:** Pairwise Wilcoxon signed-rank test results for Extra Tree versus the competing models.

Compare	MeanDiff (acc)	MedianDiff (acc)	WinRate best (%)	Wilcoxon stat	p-value	HolmReject 0.05
Extra Tree vs Bagging RF	0.049149	0.048394	100.0	0.0	$7.502427 \times 10^{-10}$	True
Extra Tree vs KNN	0.021042	0.020182	100.0	0.0	$7.516019 \times 10^{-10}$	True
Extra Tree vs Random forest	0.033950	0.034288	100.0	0.0	$7.519421 \times 10^{-10}$	True
Extra Tree vs LightGBM	0.019983	0.019965	100.0	0.0	$7.526228 \times 10^{-10}$	True
Extra Tree vs AdaBoost RF	0.039167	0.039062	100.0	0.0	$7.527931 \times 10^{-10}$	True
Extra Tree vs XGBoost	0.043750	0.042752	100.0	0.0	$7.527931 \times 10^{-10}$	True
Extra Tree vs CatBoost	0.048203	0.049913	100.0	0.0	$7.543271 \times 10^{-10}$	True
Extra Tree vs Bagging LR	0.481085	0.480903	100.0	0.0	$7.544977 \times 10^{-10}$	True
Extra Tree vs Decision tree	0.298160	0.294054	100.0	0.0	$7.546683 \times 10^{-10}$	True
Extra Tree vs Logistic regression	0.481033	0.481337	100.0	0.0	$7.548390 \times 10^{-10}$	True

The first stage of optimization of the activity recognition system is to optimize machine learning through hyperparameter tuning with the help of Bayesian optimization. Tables 4 and 5 show the performance of the single and ensemble classifiers, respectively, with this setting. The hyperparameter tuning significantly improves the performance of all models. The KNN achieves 86.89% of accuracy compared to 68.53% found in Table 1, thereby improving the accuracy by 18.36%. The increase in accuracy is found for other single classifiers as well. Among the ensemble classifiers, the improvement in accuracy due to the Bayesian optimization ranges from 0.04% to 11.8%. The LGBM achieves the highest improvement, rising from 76.35% to 88.15%. However, the extra tree still performs best; it achieves an accuracy of 89.54%, which is 2.74% higher than that obtained without optimization. Another important observation is that the precision of all classifiers increases more than the improvement in accuracy. This indicates that the activity detection rate has increased. The precision of most classifiers is very close to accuracy. This development comes from the improvement in FNR. Furthermore, the gap between recall and specificity has reduced considerably, thereby reducing the bias of the classifiers toward a specific class.

The impact of feature selection on the accuracy will be investigated next. From the above discussion, it is evident that the single classifier underperforms all ensemble classifiers. For this reason, we will consider the ensemble classifiers only for further investigation. There are two-fold impacts of feature selection methods on the performance of ML models: accuracy and training and testing times. Now, we will investigate the impact on accuracy, while the latter one will be evaluated later. We employ five feature selection methods, such as variance threshold, extra tree-based method, recursive feature elimination, mutual information-based, and L-1 based methods. Table 6 shows the percentage of accuracy of various ensemble classifiers after applying the feature selection methods. Compared to Table 5, the application of feature selection leads to mixed effects on accuracy. Five classifiers, such as extra tree (ET), random forest, bagging (RF), adaboost (RF), and XGB, experience an improvement in accuracy, while the rest experience a degradation. The highest accuracy of 90.54% is found for the extra tree due to the RFE feature selection method, an improvement of 1%. Due to the improvement of 1.05%, the accuracy obtained by the random forest reaches 86.94%. The bagging with the RF base classifier benefits most due to the extra-tree based feature selection method; it achieves 3.29% improvement, which brings it to 85.59%. On the other hand, none of the feature selection methods brings any improvement in the performance of bagging (LR), catboost, and LGBM. The highest degradation is experienced by LGBM, whose minimum performance degradation is 4.56%. It is also observed that the extra tree-based feature selection method contributes to producing the best (ET), second-best (RF), and fourth-best (bagging (RF)) classifiers. RFE provides the highest accuracy of four models: bagging (LR), adaboost, catboost, and XG-boost classifiers. The mutual information, on the other hand, provides the highest accuracy for the LGBM model only.

As the hyperparameters of machine learning models play a crucial role in the effectiveness of the models, we apply Bayesian optimization to find the optimal set of hyperparameters. Table 3 shows the optimized hyperparameters of the machine learning models. The performance of various optimized machine learning models on the optimized dataset, due to the feature selection, in detecting activity, is shown in Table 7. The hyperparameter optimization results in the improvement of up to 5.26% in accuracy for the XG-boost, with the minimum improvement of 0.22% obtained for bagging (RF). This significant improvement in the XG-boost model’s performance makes it the second-best model, after the extra tree, which consistently improves performance in each step to ultimately reach 91.41%. The improvement in the accuracy of activity recognition due to the feature selection and hyperparameter optimization ranges from 3.3% to 12.5%. The most beneficial of this processing is LGBM, while the lowest one is bagging (LR). Under the optimized hyperparameters, the RFE turns out to be the best feature selection method for activity recognition by providing the highest accuracy for all machine learning models except RF, which achieves its best performance of 87.63% accuracy with the extra-tree based feature selection. Extra-tree-based feature selection is found to be the second-best feature selection method. Figure 3 shows the impact of the proposed feature selection and hyperparameter optimization on the accuracy with respect to the default hyperparameters with all features. In this experiment, extra tree classifier turns out to be the best classifier for detecting motor imagery activity recognition from EEG signals. The probable reason behind this can be explained as follows. EEG signals generally suffer from low SNR, and motor imagery EEG signals suffer even more from the low SNR. Additionally, the EEG signals are contaminated with various artifacts, which makes the signal even more noisier. Tree-based classifiers like decision tree and random forest perform splitting based on the impurity measures. However, they are often prone to overfitting in the presence of noise, as they tend to chase small random fluctuations that appear predictive but are not (Tables 16, 17, 18).

**Table 15:** Training and testing times for L1-based feature selection.

Classifiers	All features		With FS		With HPT and FS	
	Train time (s)	Test time (ms)	Train time (s)	Test time (ms)	Train time (s)	Test time (ms)
Logistic regression	44.91	40.0586	1.1094	2.109	21.9002	0.9641
KNN	0.0070	279.027	1.00016	166.1038	1.0008	426.9654
Decision tree	1.9967	2.0579	2.0662	3.026	1.081	1.0025
Extra tree	4.7546	117.506	4.5290	107.056	10.502	88.801
Random forest	12.8526	44.938	26.158	93.0201	41.891	141.6654
Bagging (LR)	28.6481	15.061	61.3188	107.604	224.089	117.0015
Bagging (RF)	76.0132	376.089	18.77	1575.092	27.331	495.5282
Ada boost (RF)	53.3807	308.815	41.5623	258.10	19.719	1079.6589
Cat boost	38.5034	90.051	29.5034	40.198	46.928	52.00052
LGBM	1.3359	18.919	16.2304	178.415	13.852	123.000
XG-boost	3.8915	41.093	7.8315	20.141	9.382	31.0006

**Table 16:** Training and testing times for variance threshold-based feature selection.

Classifiers	No FS		With FS		With HPT and FS	
	Train time (s)	Test time (ms)	Train time (s)	Test time (ms)	Train time (s)	Test time (ms)
Logistic regression	44.91	40.0586	0.204	0.0001	1.138	1.00016
KNN	0.0070	279.027	0.0010	54.7242	0.001038	337.2204
Decision tree	1.9967	2.0579	1.107	0.00001	0.771	0.999
Extra tree	4.7546	117.506	1.726	48.7794	9.868	101.1199
Random forest	12.8526	44.938	8.913	32.7355	3.6491	182.2173
Bagging (LR)	28.6481	15.061	1.700	7.512	61.464	70.7020
Bagging (RF)	76.0132	376.089	51.285	347.4755	33.607	617.4607
Ada boost (RF)	53.3807	308.815	8.786	34.8279	16.505	1140.93565
Cat boost	38.5034	90.051	19.83	40.6558	34.822	36.6005
LGBM	1.3359	18.919	0.882	16.0369	11.247	132.91382
XG-boost	3.8915	41.093	1.722	5.00679	5.676	21.0366

**Table 17:** Training and testing time for tree-based feature selection.

Classifiers	No FS		With FS		With HPT and FS	
	Train time (s)	Test time (ms)	Train time (s)	Test time (ms)	Train time (s)	Test time (ms)
Logistic regression	44.91	40.0586	0.369	1.0089	1.489	2.0017
KNN	0.0070	279.027	0.0021	84.098	0.00196	517.2445
Decision tree	1.9967	2.0579	1.04	0.9992	0.781	1.538
Extra tree	4.7546	117.506	2.378	81.2604	8.633	89.1444
Random forest	12.8526	44.938	11.269	35.548	30.575	120.16963
Bagging (LR)	28.6481	15.061	2.421	9.3138	43.794	65.85168
Bagging (RF)	76.0132	376.089	54.145	622.2720	25.833	675.2543
Ada boost (RF)	53.3807	308.815	9.433	47.7852	15.505	1047.3339
Cat boost	38.5034	90.051	22.348	41.599	35.130	32.5317
LGBM	1.3359	18.919	0.944	19.0348	12.645	141.5998
XG-boost	3.8915	41.093	2.722	9.521	7.102	23.0417

**Table 18:** Training and testing time for mutual information-based feature selection.

Classifiers	No FS		With FS		With HPT and FS	
	Train time (s)	Test time (ms)	Train time (s)	Test time (ms)	Train time (s)	Test time (ms)
Logistic regression	44.91	40.0586	306.9984	1.0008	1619.24052	0.9651
KNN	0.0070	279.027	2.0005	244.0004	2.5129	244.0004
Decision tree	1.9967	2.0579	1268.0699	1.0004	822.7841	0.9577
Extra tree	4.7546	117.506	2173.6185	55.00078	9367.70415	89.6818
Random forest	12.8526	44.938	10949.7683	32.999	36671.6465	119.4078
Bagging (LR)	28.6481	15.061	2465.2693	13.0009	64764.6229	94.4035
Bagging (RF)	76.0132	376.089	61189.893	386.9981	29027.3966	621.824
Ada boost (RF)	53.3807	308.815	10820.557	35.00008	19847.3920	1083.65273
Cat boost	38.5034	90.051	25626.3809	46.004	41403.4419	43.0464
LGBM	1.3359	18.919	1181.9295	18.0032	13504.3759	100.83484
XG-boost	3.8915	41.093	2361.4683	8.0025	7000.440	18.5308

**Table 19:** Training and testing times for RFE feature selection.

Classifiers	No FS		With FS		With HPT and FS	
	Train time (s)	Test time (ms)	Training time (s)	Test time (ms)	Training time (s)	Test time (ms)
Logistic regression	44.91	40.0586	0.222	0.00001	1.236	1.6918
KNN	0.0070	279.027	0.0025	48.2172	0.00412	319.0892
Decision tree	1.9967	2.0579	1.148	1.0061	0.658	0.99231
Extra tree	4.7546	117.506	1.834	46.319	6.502	91.531
Random forest	12.8526	44.938	10.608	34.02471	25.124	122.18619
Bagging (LR)	28.6481	15.061	3.184	15.5098	42.899	53.37648
Bagging (RF)	76.0132	376.089	51.117	28.1119	23.308	707.1619
Ada boost (RF)	53.3807	308.815	11.117	38.111	14.667	1058.1803
Cat boost	38.5034	90.051	25.945	50.6122	31.334	6.0002
LGBM	1.3359	18.919	1.201	17.6384	9.793	103.054
XG-boost	3.8915	41.093	3.358	5.5119	5.778	30.997

Consequently, they fail to effectively identify the underlying patterns in brain signals. In contrast, the extra tree does not go for the optimal split. Instead, it incorporates randomness in both feature selection in each node and threshold selection in each splitting. This makes each tree very weak and noisy on its own. Nevertheless, when hundreds of uncorrelated trees are averaged, the random noise cancels out and only the true pattern that appears consistently across trees survives in the ensemble output. This randomness acts as a regularization mechanism, which eventually improves the generalization performance on noisy EEG signal.

**Fig. 10:**
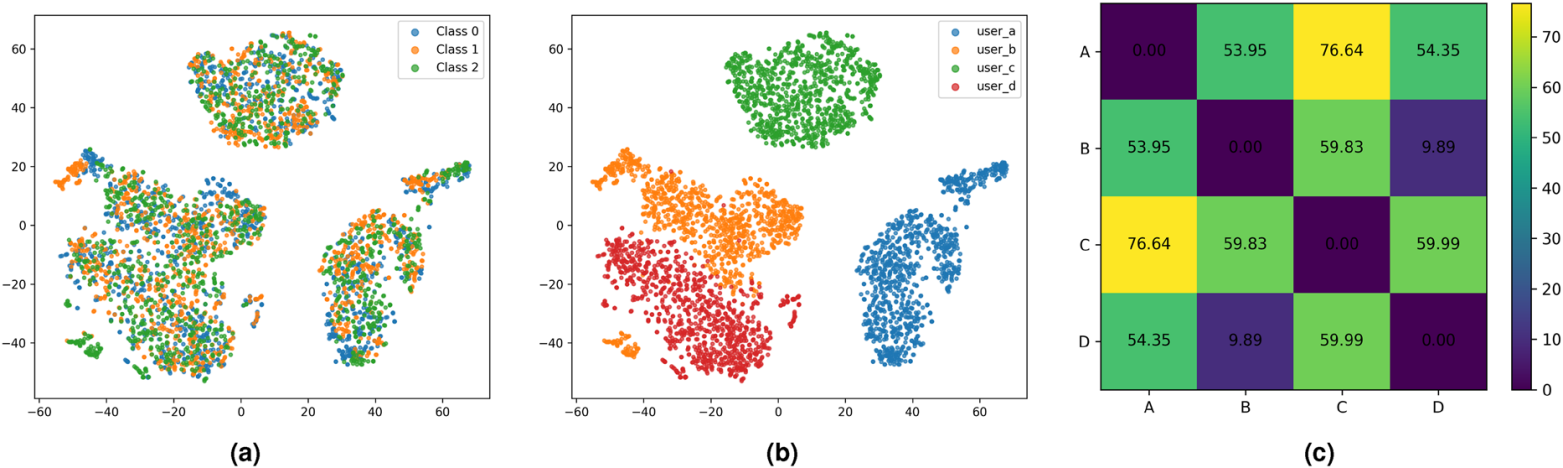
Inter-subject structure analysis: (**a**) t-SNE projection colored by subject, indicating dominant subject-specific clustering, (**b**) t-SNE projection colored by class, indicating comparatively weaker class-level separability, (**c**) pairwise CORAL-distance matrix, highlighting covariance shift across subjects.

**Fig. 11:**
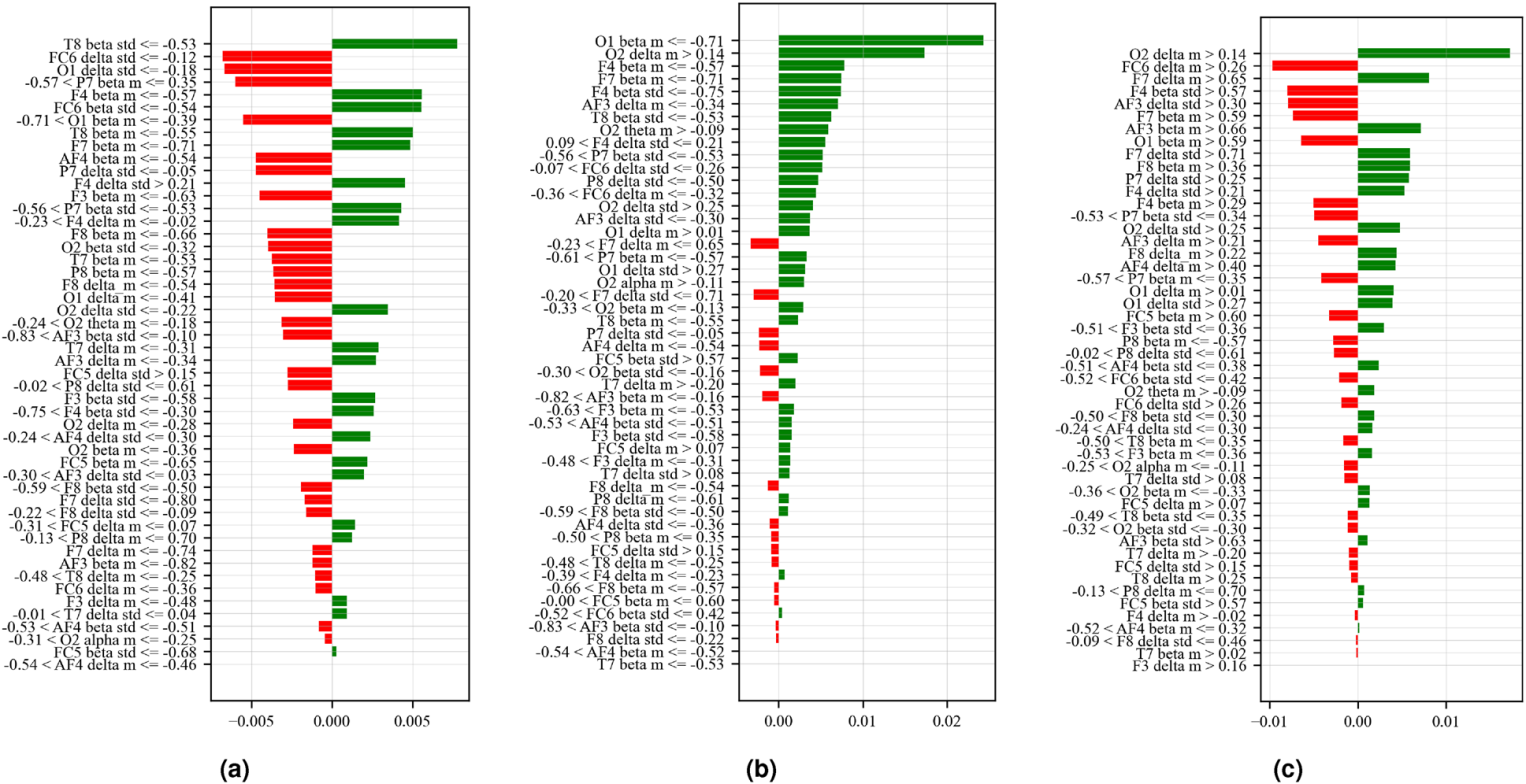
LIME local explanations for the three classes: (**a**) class 0, (**b**) class 1, (**c**) class 2.

To investigate the performance of the proposed method, optimized extra tree with RFE, we consider^19^ as the baseline method, as this is the only work done on the EEG-HM dataset, in which GRU and LSTM are employed as classifiers. Additionally, the performance of this model is also investigated with respect to a popular dataset, PhysioNet^23^. The proposed method is applied independently to both EEG-HM and PhysioNet datasets. This approach evaluates the generalizability of the proposed system across datasets with differing characteristics, ensuring no cross-dataset interaction or bias while demonstrating robust motor activity recognition performance. The following recently published, most relevant works are also considered as baselines: adaptive deep belief network (ADBN) optimized with far and near optimization (FNO)^63^, gated parallel feature fusion multi-task learning (GPFMTL)^64^, SVM with self attention (SVM-SA)^65^, LSTM^66^, and neural network (NN)^67^. The comparison is shown in Table 8. The proposed method outperforms the existing work by a big margin. On the EEG-HM dataset, it outperforms the GRU model by 21.47%. This model also demonstrates superior performance on the PhysioNet dataset, achieving 4.67% higher accuracy compared to state-of-the-art methods. To further strengthen the benchmark, we additionally evaluated representative DL models, namely 1D-CNN, Residual-MLP, and BiLSTM; the results are shown in Table 9. Among the models, 1D-CNN achieved the best performance with an accuracy of 77.60%, followed closely by Residual-MLP with 77.47% accuracy, while BiLSTM obtained the lowest accuracy of 70.14%. However, all deep learning models remained substantially below the proposed extra tree with RFE model, which achieved 91.88% accuracy on the same dataset. These results suggest that, for the EEG-HM dataset, the proposed detection method is more effective than the tested deep learning baselines. We also evaluated an autoencoder-based feature generation baseline. Specifically, latent features were learned from the provided 112 precomputed EEG features and then used as input to standard classifiers. As shown in Table 10, the best performance with autoencoder-derived features was obtained by KNN with an accuracy of 80.73%, followed by extra tree with 79.60%. However, these results remained below the proposed extra tree with RFE. To further verify that the observed performance differences are statistically meaningful, we conducted repeated experiments and summarized the results using descriptive statistics and also performed the Friedman test and the Wilcoxon signed-rank tests with Holm correction (see Tables  12, 13, 14). Table 12 shows the mean accuracy, standard deviation, and 95% confidence interval (CI) of each model. The proposed extra tree with RFE model achieves an accuracy of 91.70%, which is the highest among all models. At the same time, its standard deviation is 0.56, which shows that the model is statistically stable. The LGBM, KNN, and random forest exhibit nearly identical performance, with accuracies ranging from 88.31% to 89.70%. In Table 13, a Friedman test has been performed on all evaluated models to see whether the overall performance differences are statistically significant. Here Chi-square = 479.134510 and p-value = $1.26486 \times 10^{-96}$, which is extremely small and far below 0.05. This means that the observed difference in performance across all models is not due to random fluctuation; rather, there is an overall statistically significant difference. In Table 14, the proposed model is compared with each competing model using the pairwise Wilcoxon signed-rank test. The p-value in each comparison is on the order of $10^{-10}$, i.e., well below 0.05 in all cases, which suggests that the improvement of extra tree over each competing model is statistically significant. In addition, HolmReject_0.05 = True was found in all comparisons, which remains significant even after multiple comparisons correction.

From the above discussion, we see that the RFE is the best feature selection method for activity recognition. Next, the selected features by the RFE will be investigated further. Fig. 4 shows the impact of the number of selected features on the accuracy of the extra tree classifier. Only one feature can yield about 35% accuracy. Then, an iterative increase of the number of features leads to a sharp increase in the accuracy until the number of selected features reaches around 20, after which the growth in accuracy reduces and reaches its peak for 50 features. Thereafter, increasing the number of features does not affect the accuracy, with a slight degradation of accuracy is observed for using more than 90 features. The feature importance of the selected best 50 features obtained by the RFE is shown in Fig. 5. The occipital, parietal, and frontal lobes are found to be the most influential in activity recognition. In other words, the visual processing area, sensory integration and spatial awareness area, and the decision-making and execution areas are playing the main role behind the activities. This result is supported by the experimental setup. In the experiment, subjects were instructed to imagine moving their hands according to the signs viewed on the screen. Thus, a specific activity requires a vision system, awareness and attention, and decision and execution. Among the EEG bands, the beta and delta bands are the most influential in activity recognition. It is also evident that the P8 electrode is the most important electrode among all. The second most important electrode is O2 of the occipital area, which is followed by the electrodes of the frontocentral area. In addition to them, the frontocentral electrode FC6 and anterior frontal electrode AF4 also play a crucial role in the activity recognition. The feature importance scores of the selected features range from 0.013 to 0.032. Apart from task-based explanations, the electrode–frequency combinations identified as important by the model reflect patterns in the engineered EEG features that are broadly consistent with prior neurophysiological findings on motor imagery. These findings should be interpreted as model-level associations rather than direct neural evidence. Notably, the prominence of beta-band features within the 13-30 Hz range during motor imagery aligns with the established phenomenon of event-related desynchronization (ERD). The dominance of frontocentral electrodes (such as FC6, F4, AF4) corresponds to the premotor and supplementary motor regions involved in motor preparation. During hand imagery, the involvement of T7/T8 supports contralateral sensorimotor organization. Although traditional MI research emphasizes C3/C4, visually guided scenarios may involve occipito-parietal regions such as O2 and P8 for visuomotor integration. Nevertheless, the prominence of occipital features should be interpreted with caution, as these regions may also reflect visual processing of the arrow cues in addition to motor imagery-related activity. Since the EEG-HM dataset was acquired under realistic consumer-grade EEG conditions and only precomputed FFT-derived band features are publicly available, direct separation of visual-evoked, ocular, and motor-related contributions was not possible in this paper. Therefore, the reported feature importance likely reflects a combination of visuomotor, attentional, and motor-intention-related processes rather than purely motor-cortex-specific decoding. In addition, the consistent selection of standard deviation features indicates that dynamic oscillatory modulation – instead of static power alone – contributes significantly, aligning with the transient properties of ERD/ERS phenomena.

**Fig. 12:**
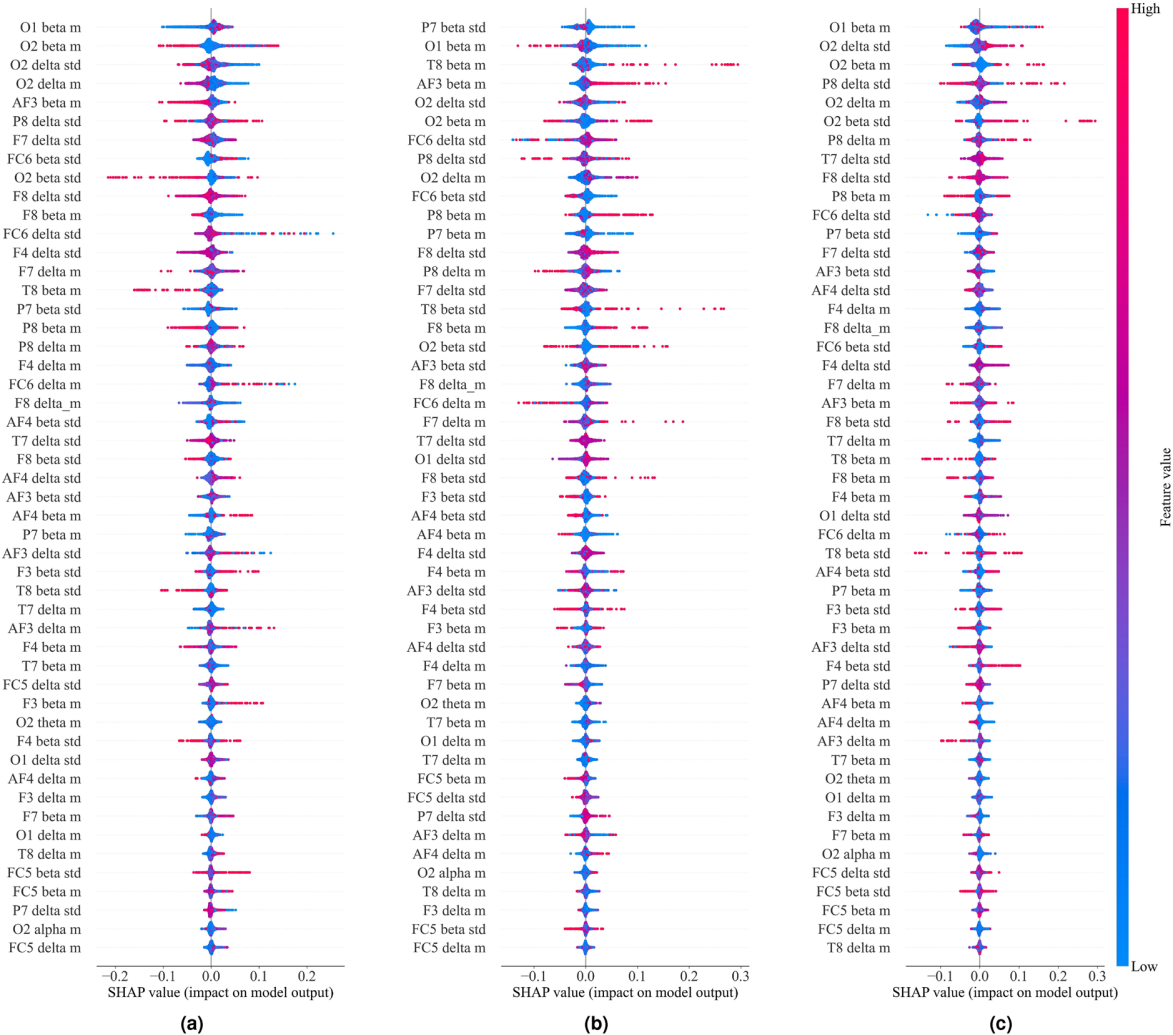
SHAP beeswarm plots for the three classes: (**a**) class 0, (**b**) class 1, (**c**) class 2.

The accuracy of a machine learning model may not properly reveal the effectiveness of the model across all classes due to its bias toward a specific class or inability to detect a specific class. To get a clear insight of a model, the confusion matrix often plays a vital role. In what follows, we will investigate the quality of the models using confusion matrix and receiver operating characteristic (ROC curves) of the machine learning models in the considered four scenarios. Figure 6a–d shows the confusion matrices of the best extra tree classifier with RFE feature selection and Bayesian hyperparameter tuning. In Fig. 6a, we see that out of the three classes, extra tree model can detect the EEG signal with no activity most accurately compared to other two activities: right-hand and left-hand motor imagery movement, respectively. The no activity EEG signal can be detected with 86.79%, the accuracies for detecting the left- and right-hand imagery movements are 84.11% and 83.58%, respectively. It is also found that EEG signal due to no activity is less distinguishable from the right-hand imagery movement compared to the left-hand imagery movement, as the proposed system faces more confusion in distinguishing no activity EEG from the right-hand imagery movement EEG. 7.24% of no activity is misclassified as right-hand imagery movement. A similar pattern is also observed for the left-hand imagery movement activity. The optimized extra tree classifier achieves a considerable improvement in classifying all activities, with the right-hand imagery movement classification gaining the most improvement of 6.68% by reaching 89.99% (see Fig. 6b). All activities can be recognized with more than 89% accuracy. This leads to a reduction of up to 4% FNR. Then, the impact of feature selection, RFE in this case, is depicted on individual class prediction is shown in Fig. 6c. A little degradation in the detection of the left- and right-hand imagery movements is found, while the percentage of the no activity recognition reaches 91.74%, which is 1.27% higher than the optimized extra tree model. Finally, amalgamation of RFE with the optimized extra tree model gives the highest accuracy of more than 91% for all classes. The left-hand motor imagery movement can be detected with the highest accuracy of 92.06%. Furthermore, the difference in detection accuracy among the activities has been reduced to a minimum among the four scenarios. This ensures the most balanced performance of the proposed motor imagery activity recognition system.

Figure 7a–d demonstrate the evaluation of the optimized machine learning models with RFE with respect to ROC. In this case, we consider macro average of all classes. In all cases, the performance of logistic regression and bagging (LR) is close to random guessing. Optimized extra tree attains the highest TPR and FPR ratio. With default hyperparameters with all features, the performance of all classifiers is consistent. However, discrepancies between them widen when applying hyperparameter optimization and feature selection independently. It is also observed that the optimization of the models causes a significant improvement in the TPR and FPR ratios. Another interesting observation is that KNN, being a single classifier, outperforms several ensemble classifiers. The selected features and optimized models improve consistency among the classifiers. In all these cases, the area under the curve (AUC) of the extra tree classifier is the highest among all. Even a single optimization, model optimization, or feature optimization, gives the highest AUC of 0.98 for extra tree classifier. When both optimizations are performed, extra tree and LGBM achieve the same AUC. Figure 8 shows the ROCs of all optimized models with RFE feature selection for individual classes. The most discrepancy among the classifiers is found in detecting the motor imagery movement of the right-hand (class 2). The AUCs of the optimized extra tree model are 0.99, 0.98, and 0.99 for the classes no activity, left-hand, and right-hand imagery movement, respectively. To further evaluate the impact of cross-validation and the possibility of data leakage, we performed RFE feature selection in each fold of cross-validation. The selected features were then applied to the corresponding validation fold. Table 11 and Fig. 9 show the fold-wise performance of the extra tree classifier with RFE under this leakage-free 5-fold cross-validation setting. It is also observed that the feature selection within each training fold does not significantly affect the overall system performance, as the achieved accuracy remains comparable to that obtained using the out-of-fold feature selection setting.

**Fig. 13:**
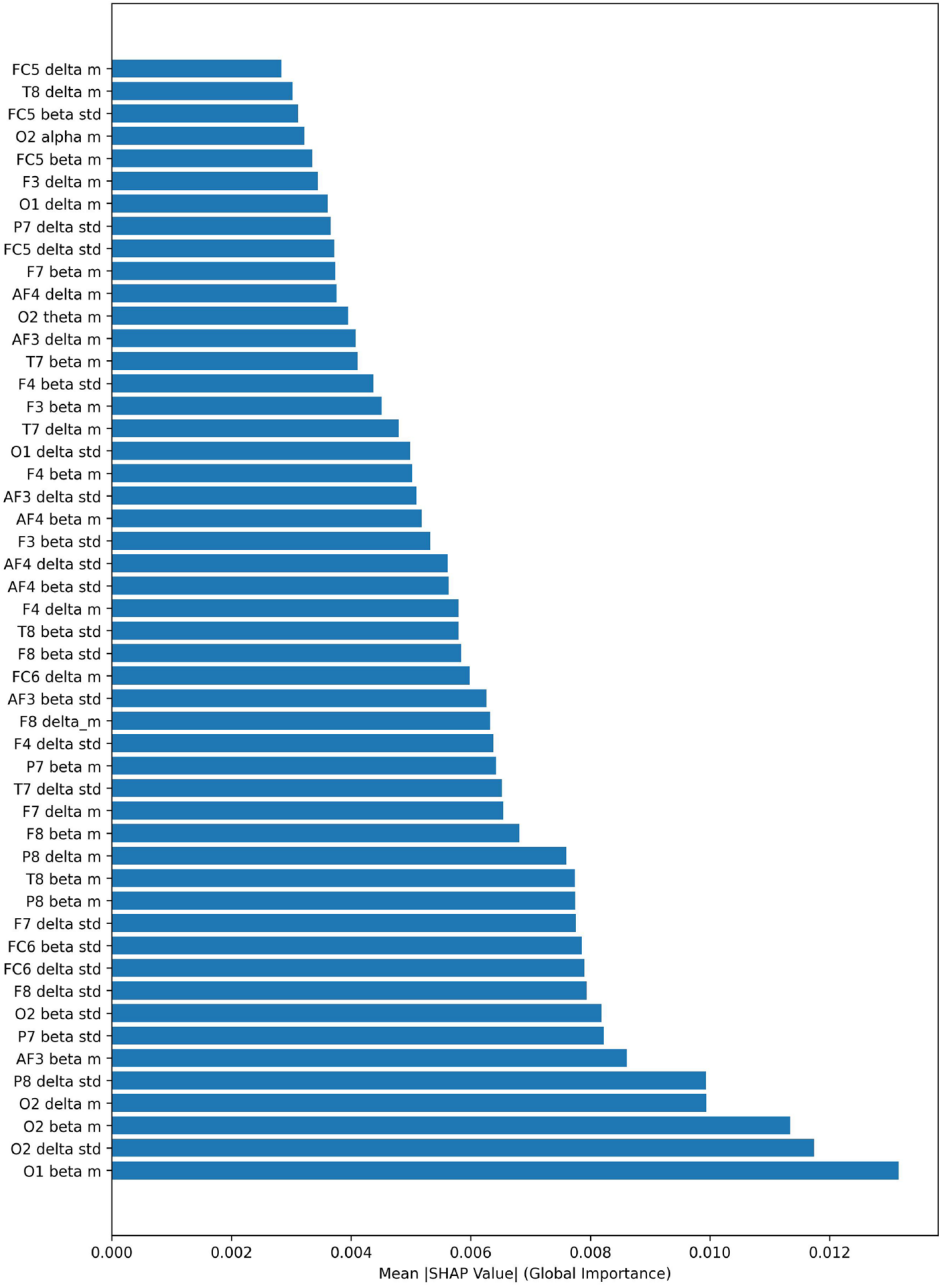
SHAP global importance.

After evaluating the machine learning models with respect to accuracy under various methods of optimization, we will evaluate them in terms of training and testing times to assess how fast they are. The models are trained and tested in a system with Intel Core i5 10 Gen 2.5GHz CPU, Nvidia GTX 1650 GPU, and 16GB RAM. Tables 15, 16, 17, 18, 19 show the impact of each of the optimization tasks on the training and testing times. For each feature selection method, three settings are considered: default hyperparameters and all features, default hyperparameters with selected feature selection, and optimized hyperparameters with selected features. The test time is measured by predicting the entire test data. It is clear from the tables that the feature selection and hyperparameter tuning have a considerable impact on the training and testing times. The feature selection method, RFE in this case, reduces training and testing times significantly for all models. For example, while the training time for extra tree and ada boost are 4.75 and 53.38 seconds, respectively, when the original dataset is used, the times reduce to 1.83 and 11.11 seconds after applying RFE. The same trend is followed for the testing time. For example, RFE has reduced testing time by more than 2.5 times for most classifiers. However, the hyperparameter optimization increases both training and testing times of all models. This can be explained as: to increase the accuracy of the models, the Bayesian optimization sets values of the hyperparameters in such a way that the accuracy increases. For example, a higher number of estimators in ensemble classifiers is used. However, this increase in the number of estimators leads to an escalation of the training and testing time. Among all the models, the decision tree is the lightest as it takes the shortest time in the training and testing phases. This is reasonable as it is a single classifier and requires very limited computations. However, it is one of the least performers in terms of accuracy. Among the classifiers that provide competitive accuracy, the LGBM and XG-Boost are the fastest models. Their training time is almost below 10 seconds, while the testing time is less than 20 ms, except for the LGBM, which requires 97 ms for RFE with the optimized hyperparameters (see Table 19). Bagging and AdaBoost with RF as the base classifier turn out to be the heaviest models. On the other hand, the extra tree model, the best model in terms of accuracy, incurs moderate computational overhead. Its training and testing time with RFE and Bayesian optimization are 6.50 seconds and 91.53 ms, respectively. Comparing Tables 6 and 7, we see that the hyperparameter optimization improves 1.6% improvement in accuracy. However, this improvement is achieved at the expense of an increase in the training and testing times by about 3.5 and 2 times, respectively. Compared to the case of all feature and default hyperparameters, while the accuracy improves by 5.38%, training time increases by 1.8 s, and the test time reduces by 26 ms.

In online MI-BCI systems, testing time is important for real-time operation since it indicates how rapidly the trained classifier can provide an output when in use. The overall testing time for the proposed extra tree with RFE model on the EEG-HM dataset was 91.531 ms for 2304 test samples, or 0.0397 ms per sample (see Table 19). Furthermore, when the same pipeline was used on the PhysioNet EEGMMIDB dataset, the average testing time was 315.218 ms for 7902 test samples, which equates to 0.0399 ms per sample. A study^68^ reported approximately 60 min training time and 5 ms/sample testing time on EEGMMIDB, which is much longer than that of the proposed method. Therefore, the proposed method appears computationally efficient and potentially suitable for low-latency discrete command-based MI-BCI applications, including assistive device control.

**Fig. 14:**
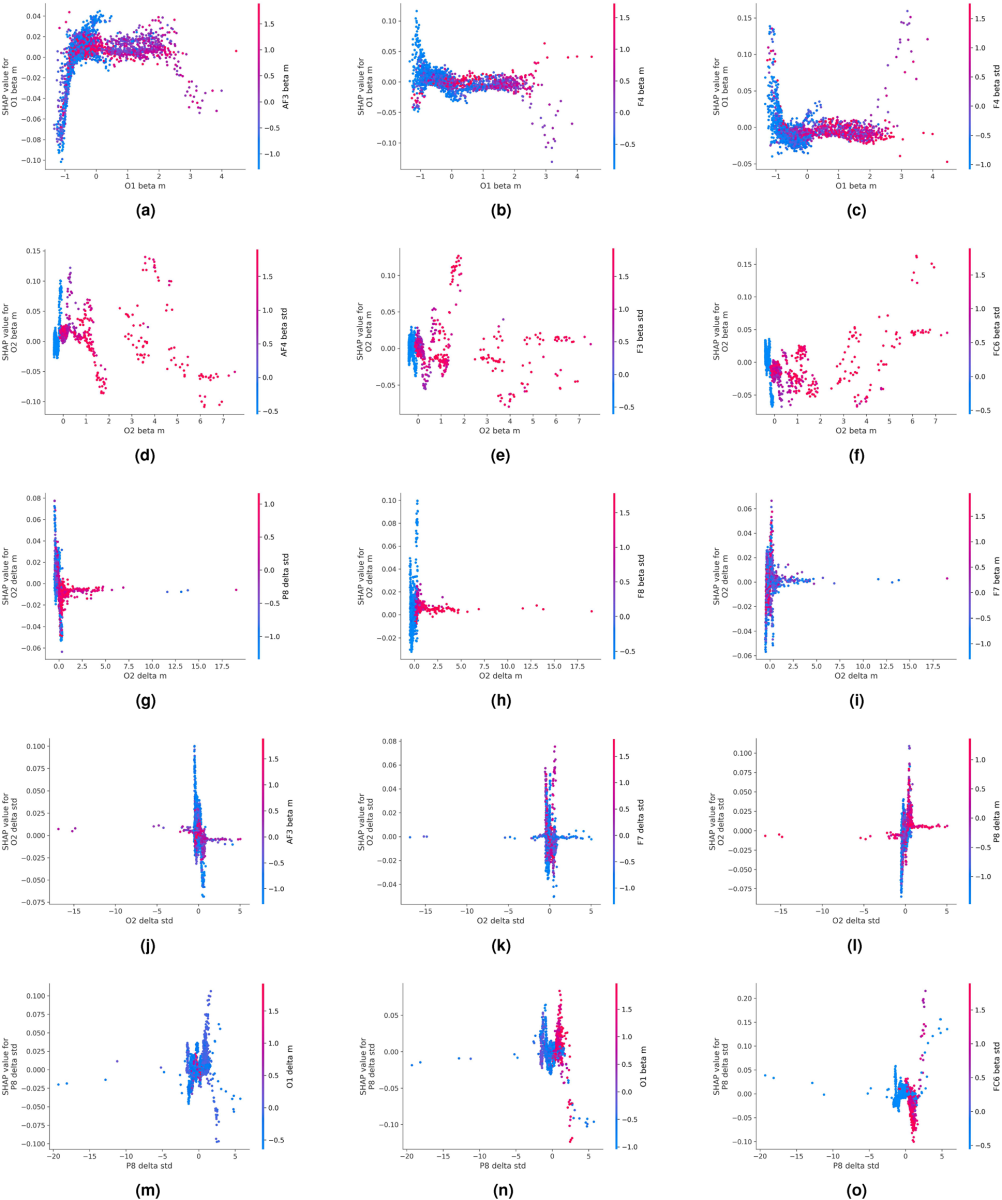
SHAP dependence plots across classes. (**a–c**) O1 beta m (classes 0–2); (**d–f**) O2 beta m (classes 0–2); (**g–i**) O2 delta m (classes 0–2); (**j–l**) P8 delta std (classes 0–2); (**m–o**) P8 delta std (classes 0–2).

**Fig. 15:**
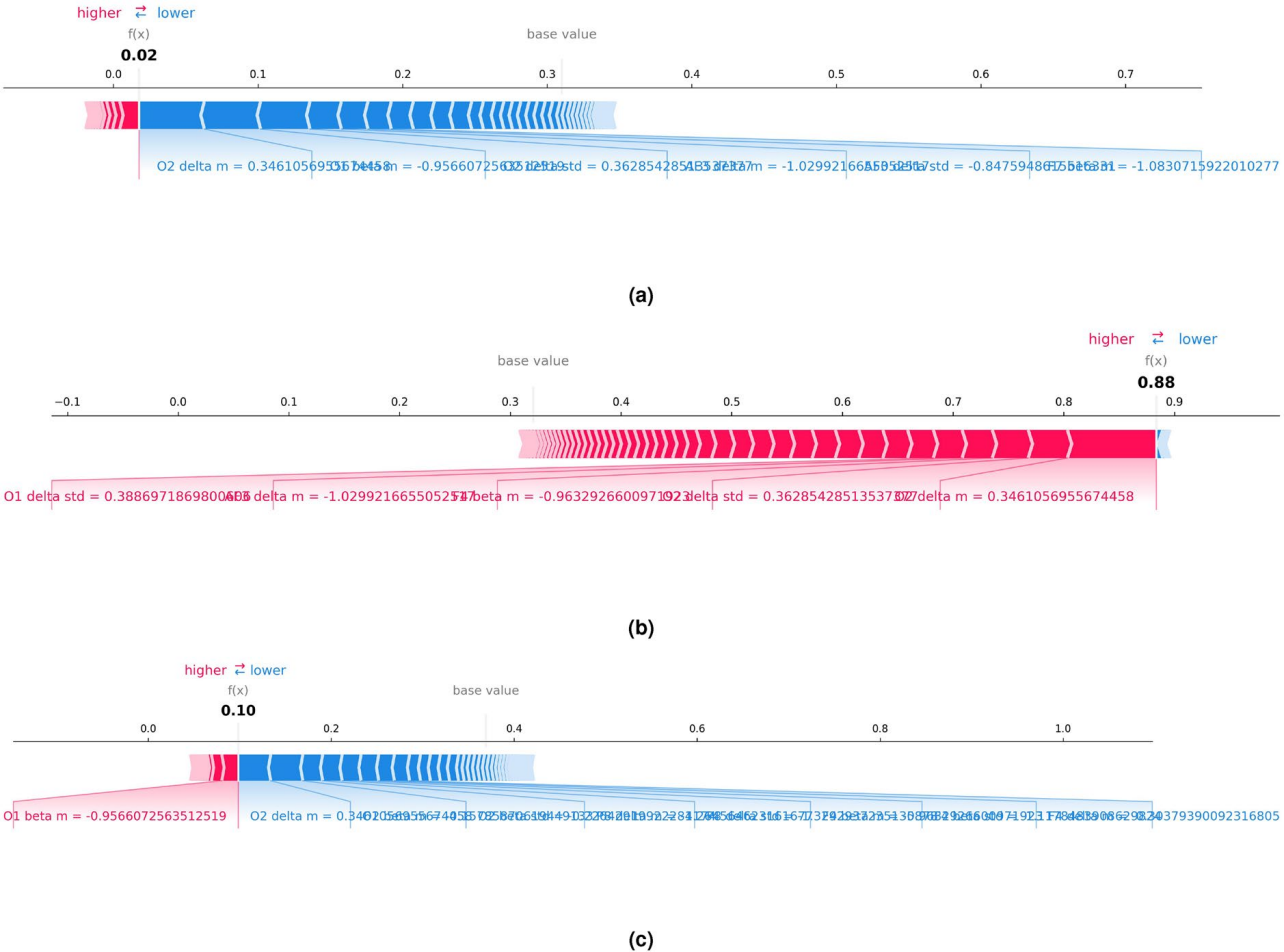
SHAP force plots for the first sample: (**a**) class 0, (**b**) class 1, (**c**) class 2.

**Fig. 16:**
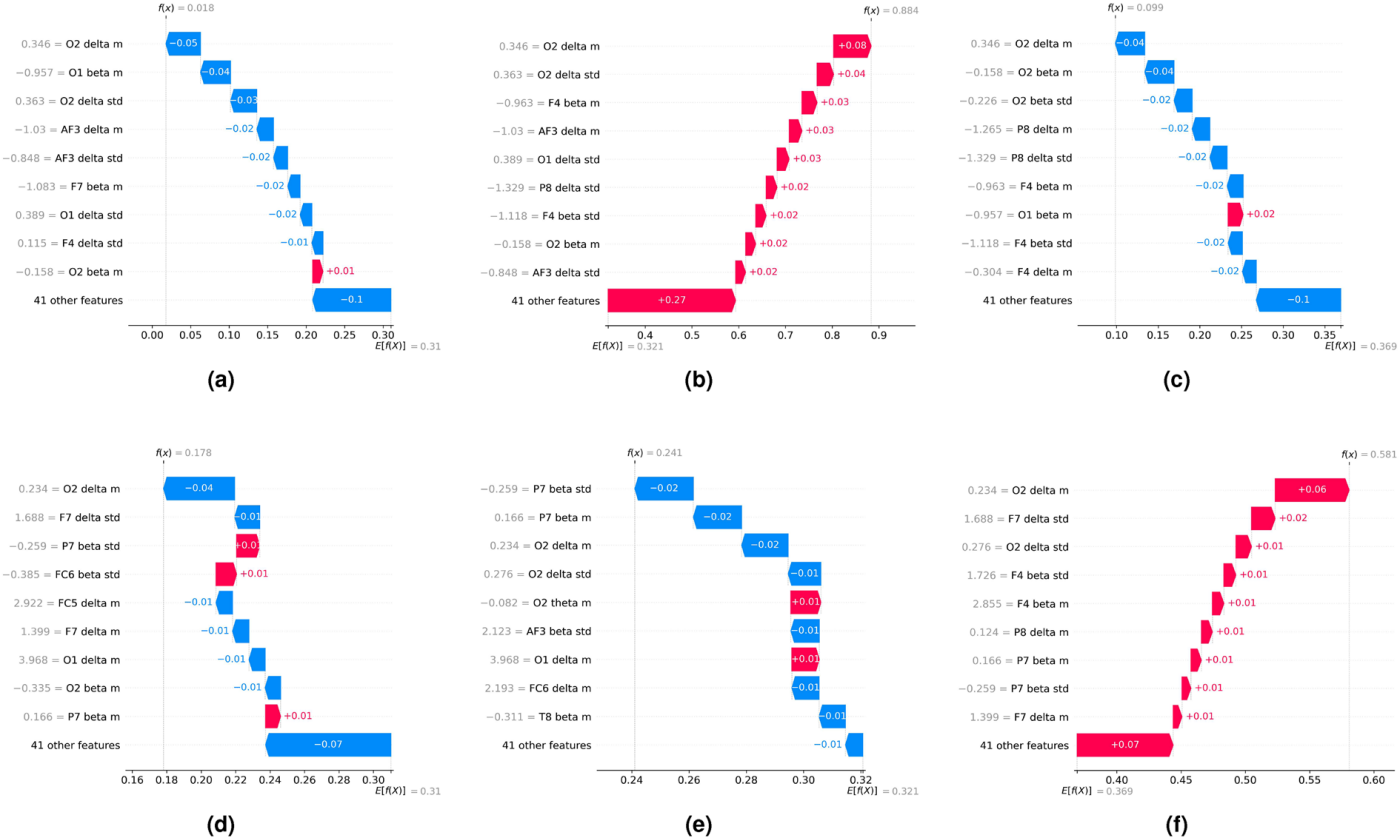
SHAP waterfall plots. (**a–c**) Sample 1 for classes 0–2; (**d–f**) Sample 2 for classes 0–2.

### Subject-independent evaluation and inter-subject variability

To assess inter-subject generalization, a strict leave-one-out (LOSO) protocol was employed to train the model on samples from three subjects and evaluate on the remaining held-out one subject in each of the four folds. Both standardization and RFE-based feature selection were applied only on the training subjects and then to the held-out subject to prevent data leakage. The proposed pipeline was used in this protocol, yielding fold accuracies of 32.50% (user_a), 40.83% (user_b), 34.41% (user_c), and 33.68% (user_d), with an overall LOSO accuracy of 35.36% and a macro-F1 score of 33.92% (see Table 20). For a three-class task, the chance level is 33.33%, indicating that subject-independent performance is only slightly above chance. This significant performance degradation in comparison to the subject-dependent environment is consistent with the established view that inter-subject variability is a substantial barrier to cross-subject generalization in motor imagery EEG^69^.

**Table 20:** Fold-wise performance of the proposed model under LOSO cross-validation [%].

User folds	Test subject	Accuracy	Recall	FPR	Specificity	FNR	Precision	F1-score
Fold-1	User_a	32.50	32.50	33.75	66.25	67.50	21.73	25.93
Fold-2	User_b	40.83	40.83	29.58	70.42	59.17	38.19	32.55
Fold-3	User_c	34.41	34.41	32.80	67.20	65.59	34.71	30.61
Fold-4	User_d	33.68	33.68	33.16	66.84	66.32	31.71	23.99

**Table 21:** Subject-wise performance of the proposed model in the subject-dependent setting [%].

Subject	Accuracy	Recall	Precision	F1-score
user_a	95.14	95.14	95.20	95.15
user_b	97.74	97.74	97.74	97.74
user_c	77.43	77.43	77.47	77.44
user_d	92.88	92.88	93.04	92.88

In contrast to LOSO generalization, the subject-dependent experiments produced good performance, with accuracy ranging from 77.43% to 97.74% across the four subjects (see Table 21). These findings show that, while the proposed model is very good at capturing discriminative patterns of the samples of individual subjects, these patterns are not sufficiently robust among unseen subjects. Consequently, the higher subject-dependent performance mainly reflects the model’s ability to learn subject-specific patterns, whereas the LOSO analysis shows a more practical view of how well the model generalizes to unseen subjects. In EEG studies, subject-based holdout is preferred for evaluating real-world applicability because sample or segment-based splitting may overestimate performance by placing subject-specific information in both the training and testing sets^70^.

To further analyze the reported LOSO degraded performance, additional FFT/wavelet feature space analysis was performed. The t-SNE visualization of samples colored by subject (see Fig. 10a) demonstrated strong close grouping based on subject identification, but the t-SNE visualization of samples colored by class (see Fig. 10b) reveals that there is significantly weaker separation available among the three motor-imagery classes. This pattern indicates that the extracted features are more strongly related by subject-specific structure than class-discriminant information. Additionally, the CORAL-distance analysis (see Fig. 10c) presents a significant covariance shift among subjects. The pairwise CORAL distance is greatest between subjects A and C (76.64) and the smallest between subjects B and D (9.89), showing significant variation in feature distributions between subjects. A supplementary approximate permutation test under a computationally LOSO configuration yielded $p \approx 0.032$ (30 permutations), where *p* is the probability, assuming the null hypothesis is true, of observing a result this extreme or more extreme. These findings suggest that the current feature representation contains subject-specific information, explaining the notable difference between subject-dependent and subject-independent performance^71^.

Overall, the current findings from above indicate that the proposed pipeline should be considered as a strong subject-dependent benchmark rather than as evidence of robust cross-subject implementation. These findings are further supported by the small cohort size (n = 4), as each LOSO fold exposes the model to only three training domains, limiting the amount of inter-subject heterogeneity available to the model for learning. As a result, the LOSO results provided here should be considered as a difficult but instructive starting point for subject-independent motor-imagery categorization on this dataset. This conclusion coincides with recent motor-imagery EEG studies^72^, which found a significant difference between subject-dependent and subject-independent performance, even when a more advanced classifier was applied on a much larger dataset with more subjects.

### Explainability of the model

In Fig. 5, the importance of various features for motor imagery activity recognition is analyzed. However, this analysis fails to identify how the frequency bands, electrodes, and the magnitude of signal levels affect the detection of specific motor imagery activity. To explore this question, explainable machine learning is exploited next. Figure 11 presents LIME-based local explanations for three classes: no activity, left-hand, and right-hand motor imagery movement. For each class, LIME identifies the most influential EEG features that contributed to the model’s prediction. Positive feature weights (green bars) indicate attributes that increased the likelihood of the corresponding class, whereas negative weights (red bars) decrease it. For left-hand motor imagery, features derived from right-hemisphere channels (e.g., F4, FC6) exhibit a dominant positive influence, while right-hand MI shows the opposite trend, with left-hemisphere features (e.g., F3, FC5, F7, AF3) contributing more strongly. These results confirm that the classifier’s decisions align with known neurophysiological patterns of motor imagery and demonstrate that LIME provides interpretable and trustworthy explanations for EEG-based classification.

Figure 12 represents the SHAP beeswarm plots for each class. The *X*-axis represents SHAP values, indicating each feature’s impact on the model’s output, and the *Y*-axis lists the top 50 EEG features selected by RFE. The color scale represents the actual feature values, where blue indicates lower and red indicates higher values, showing how variations in feature intensity influence the model’s predictions. Analysis of these SHAP plots highlights three key observations. First, occipital electrodes -specifically O1 beta m and O2 delta measures (mean and standard deviation) show the highest SHAP values for no activity and right-hand MI activity, pointing out that background and contralateral occipital activity strongly influence no motion and motion in the right-hand MI activity. For left-hand MI activity, the model depends mostly on frontal and frontocentral regions like F7 delta m, FC6 beta m, AF3 beta m, which show contralateral frontal dominance on motion in the right direction. Delta and beta frequency bands dominate the feature set among all classes, where alpha frequency contribution was not significant, which shows the importance of slow and fast oscillatory rhythms in motor control. The SHAP values show patterns broadly consistent with MI-related ERD/ERS-like modulation rather than simple broadband power shifts. Since the model relies on band-specific features, the observed SHAP patterns can be interpreted in relation to frequency-specific physiological activity. For Class 0, higher beta-related feature values in occipital channels such as O1 and O2 contribute positively to the model output, which may reflect resting or background visual cortical activity in the absence of motor imagery rather than MI-related desynchronization. For Class 1, lower beta-related feature values in posterior-temporal (P7, O1, T8) and right frontocentral regions (FC6, AF3) are consistent with beta desynchronization associated with MI, whereas higher delta-related values may reflect low-frequency synchronization or modulation. A similar trend is observed for Class 2, where lower beta-related values in occipital/parietal regions are also consistent with beta desynchronization, while higher delta-related values may indicate complementary low-frequency modulation. The relatively weak contribution of alpha features and the spatially selective importance of beta and delta features argue against a purely broadband explanation; instead, the results suggest model-level feature patterns that are consistent with physiologically relevant, band-specific activity. However, these findings should be interpreted as indirect evidence of ERD/ERS consistency rather than a direct time-locked ERD/ERS measurement. These interpretations reflect model-derived associations from precomputed features and should not be considered direct neurophysiological evidence, particularly given the limited number of subjects and the absence of raw EEG signal analysis.

LIME approximates the nonlinear classifier locally via a first-order surrogate model, yielding instance-specific feature weights. SHAP, on the other hand, decomposes the model output into additive Shapley values derived from cooperative game theory. These values are consistent across multiple predictions, making the model easier to interpret. In our results, dominant features identified by LIME (e.g., O1/O2 beta and delta bands) coincide with high-magnitude SHAP values, indicating alignment between local and global importance. However, SHAP exhibits lower variance across samples, suggesting greater stability and robustness in explaining the learned decision boundary. The additive consistency property of SHAP further ensures faithful attribution of feature contributions, making it more theoretically grounded for model interpretability in MI-EEG classification.

Figure 13 represents the global importance of SHAP feature averaged over three classes, where the *X*-axis shows the mean SHAP value and the *Y*-axis lists the 50 EEG features selected by the RFE. This plot shows how each feature impacts on extra tree classifier’s predictive results. Some important observations can be found from this analysis. First, occipital electrodes (O1, O2) lead the top ranks with O1 beta mean, O2 delta standard deviation, O2 beta mean, and O2 delta mean, achieving the highest mean SHAP values above 0.01, which indicates the activity in the occipital regions, specifically within the delta and beta frequency bands, plays an important role in classification. However, features such as FC5 delta mean, T8 delta mean, and O2 alpha mean present lower SHAP values, which express the lower impact on the model’s prediction. The majority of the less influential features come from the central frontal regions (F3, F7, FC5, AF4), specifically within the delta band. Both the mean and standard deviation of frequency bands contribute essentially to the model. The plot shows that the strength and temporal variability of oscillatory activity impact on classification performance.

Figure 14a–o represent the SHAP dependence plots for the most influential features across the three classes, showing how variations in features can impact the model’s prediction. The *X*-axis shows the raw feature value (mean or standard deviation of band power), *Y*-axis indicates the related SHAP value. The color gradient indicates the magnitude of the interacting feature, where lower values are shown in blue and higher values are shown in red, showing how interaction effects adjust the relationship between the main feature value and its SHAP impact. The analysis of the figures highlights some key points. First, O1 and O2 beta activity (Fig. 14a–f) express class-specific contributions, like high O1 beta values increase the chance of being classified as class 0, where higher O2 beta values increase the prediction of class 2. Second, O2 features (Fig. 14g–l) highly support class 2 predictions when both the mean and variability of delta power are elevated. While the same features reduce the prediction of class 1. Third, P8 delta standard deviation (Fig. 14m–o) impacts all classes, but its impact is strong on motor activity (classes 1 and 2) compared to class 0.

Figure 15a–c represent the SHAP force plots for the first test sample over the three classes, where the x-axis indicates the model’s probability space with the base value similar to the expected prediction over the dataset. Each colored bar indicates the feature’s contribution, where red bars show features that increase the predicted probability of a class and blue bars indicate features that decrease the prediction. Some key points can be observed, like for class 0, most contributing features (O2 delta mean, O1 beta mean, FC6 delta standard deviation) occur with negative SHAP effects, lowering predicted probability to 0.02. For class 1, features produce high positive contributions, specifically O1 delta standard deviation and O2 delta mean, following a high predicted probability of 0.88 and lastly for class 2, feature contributions are relatively weak, with O2 delta mean only contributing a positive influence.

Figure 16a–f represent the SHAP waterfall plots for two samples over all three classes, explaining how individual features collectively influence the prediction result. The *X*-axis indicates the prediction probability space. Features with red bars influence the prediction toward the target class (Positive SHAP contribution), and blue bars suppress the prediction. For the first sample (Fig. 16a–c), O2 delta mean, O1 beta mean, and O2 delta standard deviation made strong negative contributions for no activity and right-hand motor imagery movement. Inversely same features, along with F7 delta standard deviation and P8 beta mean, contributed positively for class 1, making a dominant prediction of right-hand movement. For the second sample (Fig. 16d–f), O2 delta mean, and P7 suppressed predictions for no activity and left-hand motor imagery movement by contributing negatively. In contrast, O2 delta mean, P7 beta mean, and FC6 beta standard deviation contributed positively for class 2 leads to dominate the probability for left-hand movement.

## Conclusion

In this paper, the first comprehensive benchmarking and explainable machine learning analysis were presented for the publicly available EEG imagery activity recognition dataset. We evaluated nearly a dozen classifiers, encompassing both single and ensemble classifiers, in combination with five feature selection techniques and optimized all models using Bayesian optimization to ensure fair comparison and robust performance estimation. For the first time, explainable ML methods were further employed on this dataset to interpret model behavior and identify the most discriminative EEG features, offering model-level insights that are consistent with known neurophysiological patterns.

Extensive experiments revealed that the extra tree model outperformed all other models. Among the feature selection methods, the RFE could select the most important features, which was demonstrated through its performance in improving the performance of almost all models. The extra tree with RFE provided the highest accuracy of 91.88%, which is more than 20% higher compared to the existing method. It correctly detected left-hand, right-hand, and idle states with accuracies of 92.06%, 89.99%, and 91.11%, and reached an AUC close to 0.99. The use of the feature selection also made the training much faster, cutting the time from 4.75 s to 1.83 s. In addition, the great performance of the extra tree with RFE is not limited to the EEG-HM dataset; rather, it outperformed existing methods on the much-studied PhysioNet dataset. The explainability of motor imagery activity recognition was analyzed through local LIME explanation, SHAP global importance, SHAP beeswarm plots, SHAP dependency plots, SHAP force plots, and SHAP waterfall plots.

A key limitation of this study is that, since the EEG-HM dataset was collected under realistic recording conditions and only FFT-derived band features are available, residual ocular and visual-process contributions cannot be completely excluded from the reported classification performance. A further limitation of this study is that, because the publicly available EEG-HM release contains only precomputed band-based features rather than raw EEG waveforms, raw-signal benchmark pipelines such as CSP + LDA could not be implemented; therefore, the comparison is limited to methods operating on the released feature representation. Accordingly, the reported results should be interpreted as a practical benchmarking reference for the publicly available EEG-HM feature representation acquired under realistic consumer-grade EEG conditions, rather than as a definitive standardized baseline for purely motor-cortex-specific motor imagery decoding.

Future research will focus on enhancing both the generalizability and ethical reliability of EEG imagery activity recognition systems. Transfer learning and domain adaptation approaches will be adopted to reduce subject-specific calibration requirements. Building on the current use of SHAP and LIME, future work will extend explainability toward causal and neurophysiological interpretation of learned patterns. Additionally, privacy-preserving machine learning frameworks—such as federated learning and differential privacy—will be explored to ensure the secure handling of EEG data. Real-time implementation and dataset expansion remain key priorities for practical and reproducible deployment.Furthermore, future work should validate the proposed framework on raw EEG recordings to enable end-to-end reproduction of the signal preprocessing and feature extraction pipeline.

## Data Availability

1. Eeg hand movement/user prediction data are publicly available on Kaggle Notebook (2025). Avaiable at: https://www.kaggle.com/code/gcdatkin/eeg-hand-movement-user-prediction/input 2. Data are publicly available on Mendeley Data: Physionet EEGMMIDB in MATLAB structure and CSV files to leverage accessibility and exploitation (Version 4; published 13 May 2024). Data are available in the following URL: https://doi.org/10.17632/dpmtgrn8d8.4
